# Tuberculosis Susceptibility and Inbreeding Depression Hinder *Ex Situ* Conservation in a Critically Endangered Rainforest Bird

**DOI:** 10.1111/eva.70286

**Published:** 2026-06-23

**Authors:** Peri E. Bolton, Dustin J. Foote, Nancy E. Drilling, Susan B. McRae, Michael D. Sorenson, Christopher N. Balakrishnan

**Affiliations:** ^1^ Department of Biology East Carolina University Greenville North Carolina USA; ^2^ Department of Vertebrate Zoology National Museum of Natural History, Smithsonian Institution Washington DC USA; ^3^ Department of Evolution, Ecology & Organismal Biology Ohio State University Columbus Ohio USA; ^4^ Sylvan Heights Bird Park Scotland Neck North Carolina USA; ^5^ Department of Fisheries, Wildlife and Conservation Biology University of Minnesota St. Paul Minnesota USA; ^6^ Department of Biology Boston University Boston Massachusetts USA; ^7^ Division of Environmental Biology National Science Foundation Alexandria Virginia USA

**Keywords:** conservation genetics, endangered species; population genomics, wildlife disease

## Abstract

Captive breeding can be a key component of species conservation strategies, but also exposes these rare species to novel environments including the pathogen landscape. The critically endangered white‐winged wood duck (WWWD) *Asarcornis scutulata* has experienced substantial population declines, local extirpations and fragmentation of its former range in Southeast Asia, making it one of the rarest birds in the world. As in other rare species, WWWD declines have led to the initiation of captive breeding programs, but these have been hampered by the WWWDs' high susceptibility to *Mycobacterium avium* , avian tuberculosis (TB). In this study we describe genome‐wide patterns of diversity to understand the WWWD's demographic and phylogeographic history, inbreeding in the wild and in captivity, and the causes of TB susceptibility. Captive birds, which originated from northeast India, are genetically differentiated from wild birds sampled in Sumatra, Indonesia, likely reflecting long‐standing phylogeographic structure. Demographic analyses revealed that long‐term (Pleistocene) population declines preceded anthropogenic declines, a pattern shared with other codistributed, forest‐dependent species. All sampled WWWD populations had extremely low genetic diversity (*π* = 0.0008–0.0015) but wild‐sampled birds retained higher Major Histocompatibility Complex (MHC) diversity, reflecting important functional diversity in the wild. Genetic diversity has eroded over time in captivity and importantly, birds with higher levels of inbreeding succumb earlier to TB infections, suggesting inbreeding depression. Finally, by comparing gene expression between susceptible WWWD and resistant redhead ducks *Aythya americana* we identify possible mechanisms of TB susceptibility. Altogether our study provides genomically‐guided objectives for future management and a cautionary tale for *ex situ* conservation.

## 1 Introduction

Captive breeding is an important component of species conservation strategies. However, husbandry in captivity poses additional challenges as these rare species are exposed to novel environments, including pathogens (Ballou 1993). These novel exposures may interact with inbreeding to hinder captive breeding success. Conservation genomics has the potential to provide not only an understanding of the demographic history of rare and threatened species but also their ability to adapt and respond to novel threats in a changing environment (van der Valk and Dalèn 2024). However, genomic efforts have mostly focused on species in the global north (Linck and Cadena 2025), leaving many equally or more imperiled species in the tropics poorly understood.

**FIGURE 1:**
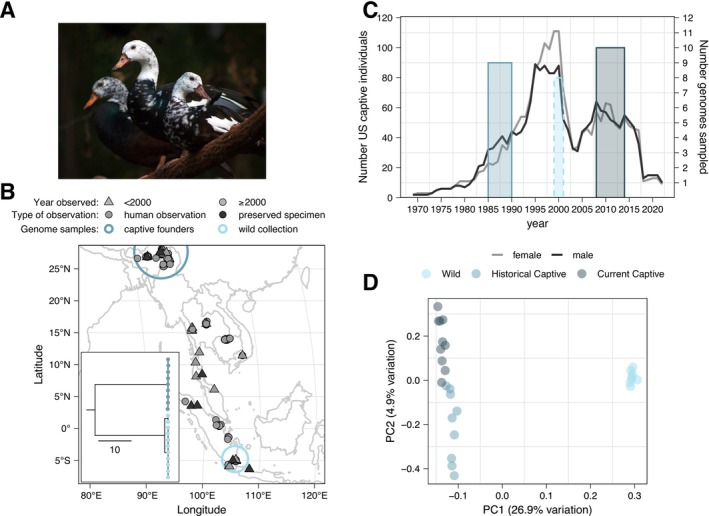
Endangered white‐winged wood ducks have experienced population declines in the US captive breeding program, and are genetically differentiated from wild ducks. (A) White‐winged wood ducks ( *Asarcornis scutulata* ) at Sylvan Heights Bird Park, North Carolina. Photo: Katie Lubbock. (B) Time series showing US captive population size of male (black lines) and female (gray lines) white‐winged wood ducks (left *y*‐axis). Colored rectangles indicate the hatch years for captive birds, and sampling period for wild birds for the genetic samples used in this study (*x*‐axis) and their height indicates the number of samples used in this study (right *y*‐axis). Three additional wild birds were included in mtDNA phylogeny. (C) Distribution of white‐winged wood ducks in Southeast Asia from Global Biodiversity Information Facility (GBIF) records (User 2024). Black points indicate museum specimens, whereas gray indicate human observations such as from eBird. Triangles show observations prior to the year 2000 indicating likely extinction in Java and Malay Peninsula. Observations in central northern Thailand are from a reintroduction program [7]. Large colored circles indicate the approximate locations of the founders of the captive population in Assam (dark blue), and the feather samples taken from wild ducks in Sumatra (light blue). Inset: Maximum parsimony mitochondrial DNA phylogeny of wild and captive birds, where scale is the number of changes along each branch. (D) Principal components analysis of autosomal variants for individuals used in this study, color coded by population.

*Asarcornis scutulata*, the white‐winged wood duck (Figure 1A), is one of the rarest birds on earth, yet we know nothing about patterns of genetic variation in this species. Also known simply as the white‐winged duck, the longer moniker is more commonly used within the species' range (Choudhury 2023) so we use that nomenclature here, abbreviated as WWWD. The current WWWD range extends from Northeast India to Sumatra, though only small, fragmented populations persist across this range (Figure 1B) (Choudhury 2006, 2023; A. Green 1992; Saikia and Saikia 2011). Somewhat unusual for waterfowl, WWWDs are forest dwelling and cavity nesting. WWWDs are highly dependent on mature rainforest with slow‐moving water bodies, habitats that have been particularly degraded over the last century. Deforestation and poaching remain the biggest threats to the survival of the species.

Recent estimates suggest only 150–450 mature adult WWWDs remain in the wild, with the largest populations in northern Myanmar and Arunachal Pradesh and Assam Provinces in India (BirdLife International 2024). The WWWD is now considered critically endangered by the International Union for Conservation of Nature (IUCN), but protection efforts in India began in 1937 and the species was placed on the Indian Special Protected List in 1952 (Mackenzie and Kear 1976). The World Wildlife Fund selected the WWWD for a focal species project in 1968. As a result, the International Waterfowl Research Bureau and the World Wildlife Fund recommended that immediate action was needed to ensure the survival of WWWD in Assam (A. Green 1990, 1992; Mackenzie and Kear 1976).

As part of a resulting conservation plan, captive populations were initiated in the United Kingdom, and then the United States. These captive populations were established using birds collected in Assam in 1969 and in 1970 (Mackenzie and Kear 1976). Both of these early wild collections took place on the same tea plantation in upper Assam (Figure 1B). Staff at two Wildfowl and Wetland Trust (WWT) centers, located at Slimbridge and Peakirk, UK, were able to rapidly grow their captive WWWD populations to a total of 86 individuals by 1976 (Richardson 1996). Soon after establishment of the captive breeding program, however, it became clear that *M. avium* —avian tuberculosis (TB)—was the source of mortality for most individuals. Between 1976 and 1991, 102 out of 121 birds (84%) succumbed to TB (Cromie et al. 1992). Originating from the same wild population in Assam, WWWDs were initially imported to various facilities in the US. Led by Sylvan Heights Bird Park (SHBP) in Scotland Neck, North Carolina, the US population expanded to just under 200 individuals (Figure 1C) but following this population growth (*circa* 1999), avian TB began to significantly affect the North American population, primarily due to an increase in mortality in birds aged 2–6 years (Cook 2016). This rapid turn of fortune for the US captive population suggests the possibility that inbreeding depression may have interacted with TB exposure to reduce the viability of captive breeding. At present, the current captive population is descended from just two wild founders.

In this study, we set out to describe genetic variation in WWWDs, including wild birds sampled in Indonesia and captive birds in the US, originally sourced from India. We aimed to provide a first look at the extant genetic diversity in this species with an aim to guide future conservation efforts. We specifically explore genetic variation in immune system genes of the major histocompatibility complex (MHC) and transcriptome‐wide expression patterns to provide mechanistic insights into TB susceptibility in WWWDs.

## 2 Materials and Methods

### 2.1 DNA Samples

Our sampling design included captive, India‐derived birds from SHBP, and wild birds from Indonesia (Table S1, Figure 1B). Captive birds were sampled as part of routine health checks or during necropsies performed by veterinary staff at SHBP. These samples represent two time periods that we label “historical” and “current.” The “current captive” sample comprised 10 individuals (six males and four females) hatched between 2008 and 2014. The historical captive sample included nine birds that were blood‐sampled at SHBP in 1994. Among the birds in the “historical” sample (*n* = 9), those with known studbook numbers hatched between 1985 and 1990 at three locations in North America; SHBP, NC; Goodewood Game Bird Farm, AL; and St. Louis Zoo, MO; all were subsequently moved to SHBP. Three individuals in the historical sample could not be associated with their corresponding studbook number and consequently have limited historical information.

Feather samples were collected from 11 wild WWWDs in Lampung Province, Indonesia, primarily in Way Kambas National Park (southern tip of Sumatra, Figure 1C), between June 1999 and February 2001 by N.E.D. Samples were exported from Indonesia with permission from the Directorate General of Forest Protection and Nature Conservation in the Department of Forestry and imported to the US under CITES permit 01US042484/9 to N.E.D. These Indonesian samples were opportunistically included. Due to the extreme rarity of these birds in the wild, however, we were not able to collect a matched sample of wild birds from the Indian population.

### 2.2 DNA Extraction, Sequencing and Read Mapping

The 10 current captive samples included DNA extracted from blood, liver, lung, and spleen. Nine current WWWD samples were extracted in 2017, with a Qiagen DNeasy Blood and Tissue kit. Historical captive WWWD DNA samples were extracted from blood samples collected at SHBP; standard phenol‐chloroform extractions were completed by MDS in 1995 and stored in a −80°C freezer before being sent to East Carolina University in 2019. The 11 wild WWWD feather samples collected by N.E.D. were sent to MDS in 2003; DNA extraction from feathers was completed using the Qiagen DNeasy Tissue and Blood kit with the addition of 30 μL of 100 mg/mL dithiothreitol to digest the feather keratin; these extracts were preserved in a −80°C freezer until being sent to East Carolina University in 2018 for whole genome sequencing.

Due to project funding and sample availability, samples were sequenced in two batches. Fragment libraries for a first batch of seven current samples were prepared with an Illumina TruSeq PCR‐free kit and sequenced at the University of Illinois Roy J. Carver Biotechnology Center on an Illumina HiSeq 4000 instrument. A second batch of 22 birds, including the remaining current, historical and wild samples, was also sequenced at the University of Illinois. These libraries were prepared with a Hyper Library construction kit from Kapa Biosystems and sequenced on a NovaSeq 6000.

For most of the population genomic analyses reported here, WWWD data were aligned to the highly contiguous tufted duck genome ( *Aythya fuligula* GCF_009819795.1, contig N50 = 17.7 Mb), but we also mapped the data to the short‐read‐based WWWD genome assembly (GCA_013398475.1, contig N50 = 91.2 kb (Feng et al. 2020)). Reads were aligned to both reference genomes using *bwa mem* v0.7.17(default settings) (Li 2013). Alignment success was similarly high for both reference genomes (WWWD reference: historical = 99%, current = 99%, wild = 94%; tufted reference: historical = 99%, current = 99%, wild = 95%). We favored the tufted duck‐aligned dataset in most downstream analyses because of our interest in multi‐copy immune gene diversity, the annotations for which are better in contiguous genomes with high Contig N50 (Driver and Balakrishnan 2021; He et al. 2021). Depending on the given analysis we used either called genotypes from *bcftools* genotype likelihoods inferred by *ANGSD* v0.911 (Korneliussen et al. 2014) and using the *ANGSD‐wrapper* (Durvasula et al. 2016). We estimated genotype likelihoods in *ANGSD* with the following parameters: ‐doGLF 3 ‐GL 1 ‐doMaf 1 ‐SNP_pval 1e6 ‐doMajorMinor 1 ‐minMapQ 30 ‐minQ 20 (Korneliussen et al. 2014). Called genotypes, based on depth‐normalized aligned data, were obtained using *bcftools* (v 1.13) *mpileup* and *bcftools call ‐m ‐f GQ* (Li 2011), and included invariant sites. VCF files were filtered using *vcftools* v0.1.15 to include only sites that were present in 70% of individuals, and excluded sites with minDP < 5 and max‐meanDP > 50. Both approaches yielded comparable results in downstream population genetic analyses, such as estimates of inbreeding and population divergence (not shown).

### 2.3 mtDNA Assembly and Phylogenetic Reconstruction

To assemble the mitochondrial genomes, we aligned short read data to an existing mtDNA assembly for WWWD (GenBank accession: MN356440) and imported the resulting reads into Geneious Prime v. 2023.2.1 (https://www.geneious.com). Given the presence of nuclear copies of mtDNA sequences in related duck species (Sorenson and Fleischer 1996), we closely examined alignments for evidence of “numts” (Sorenson and Quinn 1998). For the feather samples from the wild population (*n* = 11), there was little evidence of numts and substantial coverage of the mtDNA (> 800×), allowing inference of complete, high confidence mtDNA sequences for all samples. Results were more variable for blood samples from the captive population (*n* = 19 including the sample used for the reference genome), with coverage ranging from 22× to over 500×. Divergent numt reads were evident in all samples, but represented a decreasing proportion of reads as total coverage increased. Thus, complete mtDNA sequences could be assembled for those blood samples that yielded relatively high coverage of the mtDNA; this included 1 historical sample and seven current samples with total coverage of 85× or more. To provide an estimate of the timing of divergence of mtDNA lineages, we augmented the WWWD data with available waterfowl mtDNA genomes (Figure S1) and completed a phylogenetic analysis in BEAST (v. 2.7.7 (Bouckaert et al. 2019)) using the 12 light‐strand encoded mt protein‐coding genes. We partitioned the data by codon position, employed model averaging and an optimized relaxed clock (Bouckaert et al. 2019). Following Mitchell et al. (2014) and using identical parameters values, we time‐calibrated the phylogeny by setting a lognormal prior on the age of the dabbling duck lineage based on the middle‐Miocene fossil *Anas soporata* (Zelenkov and Kurochkin 2012).

### 2.4 Reconstruction of Historical Demography

We used *SMC++* v1.15 (Nadachowska‐Brzyska et al. 2022; Terhorst et al. 2017) to compare the demographic histories of the sampled WWWD populations. Using the VCF aligned to the tufted duck genome, we randomly chose five diploid individuals from historical and wild populations as “distinguished lineages,” each representing a distinct dataset for calculation of composite likelihoods. We then estimated population history bounded between 30 and 2e5 generations and a folded site‐frequency spectrum. We additionally explored demographic history using *PSMC* (Li and Durbin 2011). Demographic estimates from *PSMC* are impacted by variation in coverage, with lower coverage samples missing variants. Our captive birds had lower depth than our wild birds, so for these analyses we used our two highest depth captive birds and downsampled all the wild birds to match. This procedure will tend to reduce estimates of *Ne*, so our *PSMC* findings provide relative estimates of effective population size and population trajectory. For this analysis, we aligned trimmed reads to the WWWD reference genome using *bowtie v*2.5.4 and *–very‐sensitive* options, we used *Picard* v3.1.1 *MarkDuplicates* to remove PCR duplicate reads. We then called variants for *PSMC* using *samtools mpileup* (‐Q 30 ‐q 30 ‐u ‐v ‐f) for the 15 longest scaffolds and converted the output into a fastq using *vcfutils vcf2fq* (‐d 4 ‐D 80 ‐Q 30). Hilgers et al. (2025) note a common issue in which PSMC estimates unreasonably large effective population sizes in the most recent time interval under typical settings. Following their guidance we split the first atomic time interval (‐N25 ‐t15 ‐r5 ‐p “2+2+25*2+4+6”) which generated interpretable and reasonable demographic trajectories. SMC++ and PSMC results were scaled using a generation time of 3 years and a per‐generation mutation rate of 2.5 × 10^−9^ (Singhal et al. 2015).

### 2.5 Genetic Diversity, Inbreeding and Differentiation

Using called genotypes, we estimated individual autosomal heterozygosity in each population using *vcftools* (‐‐het) (Danecek et al. 2011). We estimated nucleotide diversity (*π*) for each of the three populations and pairwise *F*_ST_ among populations using 100 kb windows in *Pixy v2.0.0.beta6* (Korunes and Samuk 2021). These analyses were based on VCF files with invariant sites included.

We used *ANGSD* and ANGSD‐wrapper (Durvasula et al. 2016; Korneliussen et al. 2014) to visualize genetic differentiation with PCA and estimate inbreeding from genotype likelihoods. We estimated individual inbreeding coefficients using the expectation maximization algorithm in the ngsF module (Vieira et al. 2013). We used *PCangsd* to represent genetic data as principal components (Meisner and Albrechtsen 2018).

Finally, we estimated runs of homozygosity in the genome using *ROHan v1.0.1* (Renaud et al. 2019) directly from individual alignment files. We restricted the analysis to tufted duck autosomes longer than 10 Mb and used the following parameters: rohmu = 1 × 10^−3^, and window size = 500 kb. The genomic inbreeding coefficient (*F*_ROH_) was calculated as ∑*L*_ROH_/*L*_autosomes_ where ∑*L*_ROH_ is the total length of all autosomal ROHs in an individual and *L*_autosomes_ is the total length of the autosomal genome used in the analysis (Ceballos et al. 2018; Fu et al. 2019; McQuillan et al. 2008).

For captive birds only, we tested for associations between lifespan and observed heterozygosity, individual inbreeding coefficients and *F*_ROH_ using a gaussian generalized linear model in the R package *glmmTMB* and model checks in *DHARMa* (Brooks et al. 2017; Hartig 2024). We compared two models, one which included a parameter for genetic background of historical versus current population and one with the genetic measure alone. We did not explore additional model parameters to avoid overparameterizing the small sample size. Models were compared using AIC. Models and all plotting were conducted in *R* (V 4.4.0)(R Core Team 2025). In a larger studbook dataset we examined the decline in lifespan through time, and examined whether there were sex‐specific effects on lifespan. We used quantile regression of the median (*τ* = 0.5) due to severe model violations in parametric models.

### 2.6 Major Histocompatibility Complex

We hypothesized that immune genes could play an important role in mediating susceptibility to avian TB, and investigated variation in the Major Histocompatibility Complex that is known to play a key role in immunity broadly and TB specifically (Bettencourt et al. 2020; Witt 2023). We characterized diversity in functionally relevant peptide binding regions (PBR) of MHC classes Iα and IIβ. Our ability to accurately assess heterozygosity and MHC copy number may have been influenced by different factors depending on the reference genome we used. The WWWD genome assembly is likely incomplete for MHC genes, whereas the tufted duck genome may be divergent in copy number, such that some copies may have insufficient sequencing depth for accurate SNP calling (Figure S10). Therefore, we used two approaches to characterize MHC diversity in WWWDs. First, we re‐annotated the tufted duck genome using previously published consensus sequences of exons 2–4 derived from Anatidae MHC Class I and II PBRs (i.e., a total of six exons) (He et al. 2021). We extracted putative matching sequences with e‐values < 1e10 using *tblastn v2.12* (Camacho et al. 2009), then extracted the hit with the lowest e‐value when hits overlapped. We used Geneious Prime 2022 to visualize these sequences in the genomic context and checked for premature stop codons. We considered sequential copies of exons 2–4 as a complete copy. Using this method, we estimated that the tufted duck has nine copies of Class I and three copies of Class II, whereas the WWWD genome assembly has two and one copies, respectively. Class I estimates differ from the tufted duck NCBI annotation, which includes three genes, as many annotations contain multiple PBRs. Secondly, to ensure we did not underestimate diversity, we extracted a representative gene (all exons and introns) from the tufted duck genome and re‐mapped all previous MHC‐aligning depth‐normalized reads to the single copies representing Class I and Class II, respectively. Using *samtools depth*, we assessed potential copy number by comparing the mean aligned read depth at the single copy MHC against the average depth of reads mapped to the tufted duck chromosome that contains MHC, chromosome 33. Copy number was assessed by dividing the average depth of MHC reads aligned to each MHC class and dividing by the average number of reads for chromosome 33.

Using this alignment, we called MHC SNPs for each class separately using *GATK HaplotypeCaller* v4.2.0 (McKenna et al. 2010; Poplin et al. 2017). Because multiple MHC copies were aligned to a single pseudoreference we doubled the copy number and used this number with the *‐‐ploidy* flag. Then we filtered the VCF with the following expression: “QD < 2.0 || FS > 60.0 || MQ < 40.0 || MQRankSum < ‐12.5 || ReadPosRankSum < ‐8.0” (Caetano‐Anolles 2024). For this dataset, we estimated both nucleotide and amino acid diversity in PBR exons using custom code in R. For data aligned to the genome, we calculated nucleotide diversity using *pixy* and calculated individual heterozygosity directly from genotypes in PBR exons. We statistically compared diversity estimates using Wilcoxon rank sum tests corrected for multiple testing using the Holm method in the *ggpubr* package (Kassambara 2025).

### 2.7 RNA Samples

Birds in captive environments, including those at Sylvan Heights Bird Park, are frequently exposed to *M. avium* but many species are only rarely affected by infections (Witte et al. 2008). Given its role in disease defense, we hypothesized that immune gene expression could be an important mediator of differential susceptibility. To provide a window into susceptibility, we conducted RNAseq on whole blood from TB susceptible and nonsusceptible species (Table S2). We sampled blood from eight WWWDs in 2022, and seven redhead ducks ( *Aythya americana* ) to provide a comparison to a closely related species that is less susceptible to avian TB. During the sampling period in 2022, two WWWDs were symptomatic for mycobacteriosis, whereas none of the redhead ducks were symptomatic. We further included two opportunistic samples from 2019: one redhead blood sample with mycobacteriosis, and a paired sample without (total redhead sample size = 9). All instances of mycobacteriosis were fatal, and one WWWD was sampled approximately 2 h postmortem. This individual's death was unexpected as it did not show outward symptoms of mycobacteriosis, but necropsy revealed typical mycobacterial lesions on its organs.

Birds were sampled at Sylvan Heights Bird Park using a 23 gauge needle and syringe to draw blood from the brachial vein. Whole blood was immediately placed in RNAlater, and stored on ice until it was placed in a−80°C freezer. All animal procedures were reviewed and approved by East Carolina University's IACUC under Animal Use Protocol D351.

### 2.8 RNA Extraction, Sequencing & Read Mapping

We extracted RNA from blood samples using TriZol and the Qiagen RNeasy Kit, and all samples had RIN > 7. Libraries were prepared with TruSeq Stranded mRNA Library Prep and sequenced on NextSeq 2000 P2 Reagents (200 cycles).

We generated 28.4–42.2 million paired‐end reads per sample, retaining 89%–97% of reads after trimming. We mapped RNAseq reads to the tufted duck genome (*A. fuligula*, GCF_009819795.1) using *STAR* v2.7.11b after trimming with *fastp* v0.23.2 (Chen et al. 2018; Dobin et al. 2013). Of these reads, 84%–89% were uniquely mapped to the tufted duck genome (Figure S11). Reads mapping to each gene were counted using the *geneCounts* function in *STAR*.

### 2.9 Gene Expression

We filtered counts by removing genes with counts of ≤ 10 in at least six individuals. We identified three “potentially influential” individuals using Principal Component Analysis (using *PCAtools* ; Blighe and Lun 2024); these individuals had the smallest library sizes (Figures S11 and S12). However, two of these three individuals represented two‐thirds of our disease sample, and so we used a conservative approach to remove the outlier effect from our list of differentially expressed genes (see below) We explored differences in normalized gene expression using a negative binomial generalized linear model framework implemented in *DESeq2* v1.44.0. First, we compared gene expression between asymptomatic redhead and white‐winged ducks (gene expression ~ species), using seven redhead samples (excluding one outlier individual) and six WWWD samples. Then, we compared diseased versus asymptomatic birds in each species separately (gene expression ~ disease status), and compared the disease‐related expression patterns between species. To identify differentially expressed genes (DEGs), we applied the Wald Test and considered genes to be significant with an FDR‐corrected *p*‐value (*q*) < 0.05. Due to the small sample sizes of diseased individuals, we removed significant genes that appeared to be affected by the outliers, rather than disease status, using custom code. We refer to the FDR‐corrected *p*‐value of genes that passed this additional filter as q*.

Susceptibility in WWWD ducks is likely linked to immune gene regulation or function, and thus we focused on immune DEGs. We extracted immune‐related genes using a previously published list of immune GO terms (Silver et al. 2022), using the tufted duck GO annotations available on Genbank. We supplemented the immune gene list by including previously annotated MHC genes and searching the tufted duck reference annotations for genes relating to B‐cells, T‐cells, TLRs, immunoglobulin, interleukins, and interferons. We tested whether our candidate gene list was enriched in DEGs using the hypergeometric test function *phypher()* in R. To compare gene expression in the difficult multi‐copy MHC, we summed counts from MHC Class Iα and MHC Class IIβ paralogs into a single “gene” to include in the analysis, but most reads mapped to a single copy in the tufted duck genome so this did not change the signal.

We were also interested in other non‐immune pathways that could confer TB susceptibility. To describe these pathways, we explored enrichment of GO Biological Processes and KEGG pathways using *clusterProfiler* V4.12.6 (Yu et al. 2012). We considered the full DEG list (*q* < 0.05) as the foreground and the whole blood transcriptome as the background. For KEGG enrichment, we converted the NCBI gene‐ids to functional ortholog ids (“ko”) using the *KEGGREST* v1.44.1 package (Tenenbaum 2017), and used *enrichKEGG()* to identify enriched pathways. We excluded “significant” enrichments that included ≤ two genes.

## 3 Results

### 3.1 Whole Genome Sequencing

We sequenced individuals from a time series of North American captive and wild Indonesian white‐winged ducks (Figure 1B). Overall mapping rates were 97.63% to the *A. fuligula* assembly, marginally higher than mapping to the less contiguous WWWD assembly. Captive birds were sequenced to an average of 10.5× depth and wild birds were sequenced to an average of 16.5× coverage; we downsampled to equal read‐depths as needed for specific analyses (see methods). All resequencing data have been deposited in the NCBI Short Read Archive under PRJNA1258233. Three wild samples were removed from genome‐wide analyses due to poor data quality (mapping rates < 50%).

### 3.2 Indonesian and Indian Birds Are Genetically Distinctive

Analyses of both mitochondrial DNA (mtDNA) and genome‐wide variation indicate clear, historical divergence of the Indian‐derived, captive population and the wild Indonesian population. Mitochondrial lineages are reciprocally monophyletic, with 46–48 differences (~0.28% divergence; Figure 1C) between the captive and wild lineages. A time‐calibrated phylogeny (Figure S1) suggests that these lineages diverged during the late Pleistocene at ~170,000 years ago (95% HPD range = 103–241 kya). We note that this estimate is considerably more recent than the calibration point, such that it likely overestimates the actual divergence time to some degree (Ho et al. 2005). Similarly, Principal Components Analysis of genome‐wide variants using PCAngsd clearly separated the three sampled populations (Figure 1D). Captive (India‐derived) and wild (Indonesian) birds separated along PC1 (26.9% of variation), whereas the historical and current samples of captive birds separated along PC2 (4.9% of the variation) (Meisner and Albrechtsen 2018). This separation in PC space corresponds to moderately high autosomal *F*_ST_ values between captive and wild birds (historical versus wild = 0.3, current versus wild = 0.4) and relatively low genome‐wide differentiation between current and historical captive birds (0.04).

### 3.3 Genetic Diversity Is Low and Inbreeding Is High

**FIGURE 2:**
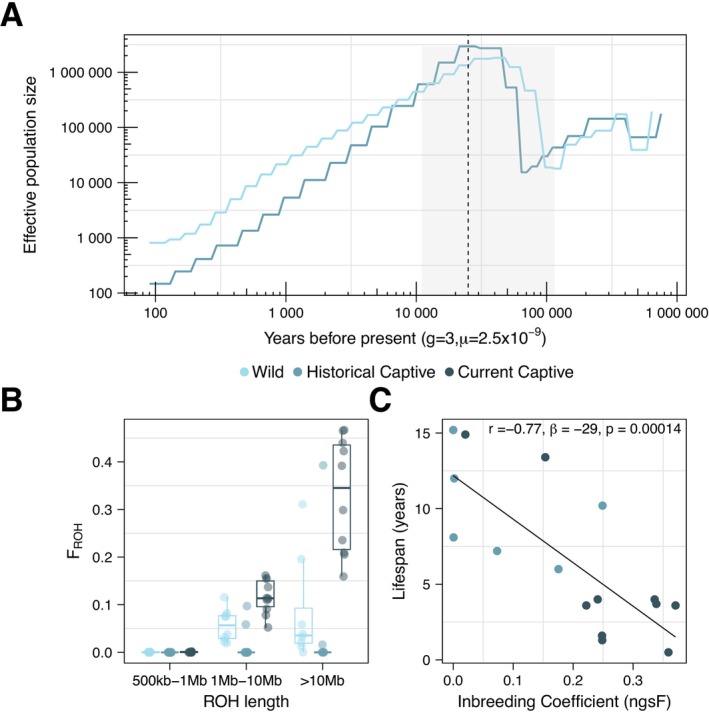
Populations of white‐winged wood ducks from India and Indonesia have declined since the Pleistocene, and have experienced ongoing inbreeding and inbreeding depression. (A) Estimates of effective population size through time from SMC++ for wild (Indonesia) and historical (India) captive ducks. Gray shaded box shows the late Pleistocene period (11.7–115 kya), with years scaled by generation time of 3 years and a per‐generation mutation rate of 2.5 × 10^−9^ (Singhal et al. 2015). (B) Inbreeding coefficients calculated from runs of homozygosity (ROH) in genomic size‐classes per individual. The line is median, the box is the interquartile range (Q1–Q3) and whiskers are +/− 1.5*interquartile range (C) Inbred birds in captivity typically live shorter lives with plotted trendline (see also Table S3 and Figures S5–S8).

Demographic analyses suggest long‐term population declines in WWWDs. Both methods (PSMC and SMC++) and both sampled populations suggest population declines since the last glacial maximum (~25 k years ago, Figure 2A). PSMC results were broadly similar to those from SMC++ (Figures 2 and S2) with minor differences among populations that may be attributed to reduced power in PSMC analyses, in which each sample is analyzed independently.

Consistent with both longer term and more recent, anthropogenically‐driven declines, nucleotide diversity (*π*) was very low in all three population samples, with current captive birds (median *π* = 0.0008) harboring about 53% of the diversity of their captive ancestors (median *π* = 0.0015). Surprisingly, genetic diversity in the wild‐sampled birds was similarly low (median *π* = 0.0010) (Figure S3). Autosomal heterozygosity estimates showed similar trends, with the wild population having lower heterozygosity than the historical captive population and the current captive population declining considerably in heterozygosity (~50%) (wild het = 0.001, historical het = 0.002, current het = 0.001; Figure S3). Consistent with patterns of nuclear diversity, a single mtDNA haplotype is found in the captive population, whereas the wild sample included two closely related haplotypes (Figure 1B). These patterns are also reflected in the lengths and abundance of runs of homozygosity (ROHs), which reflect inbreeding (Díez‐Del‐Molino et al. 2018). Recent inbreeding produces longer segments, which may be broken into shorter segments by subsequent recombination. Current captive birds had the largest number of long (> 10 Mb) segments and highest inbreeding coefficients as measured by *F*_ROH_ (*F*_ROH_ = Σ*L*_ROH_/*L*_autosomes_ = 0.47 median for the current captive population), whereas ROHs were observed in only two historical captive individuals (median *F*_ROH_ = 0, Figure 2B). Some of the wild WWWDs had many long ROHs, indicating recent inbreeding, but with a lower level of inbreeding overall (median *F*_ROH_ = 0.11). This is in broad agreement with individual inbreeding coefficients from *ngsF* (Figure S4), which were very low in the historical captive sample (median *F* = 0.007), and much higher in contemporary captives (median *F* = 0.25). In contrast, wild birds had the highest individual inbreeding coefficients (median *F* = 0.36), primarily due to intermediate length ROHs, which may indicate ongoing inbreeding in a smaller and declining population on the island of Sumatra.

For a sample of 16 captive birds with known age at death (10 current and 6 historical captives), we observed a strong and significant negative relationship between the *ngsF* individual inbreeding coefficient and age at death (Figure 2C; *r* = −0.8, *β* = −29, *p* = 0.00014, Figure S5, Table S3; see also *F*_ROH_ Figures S6 and S7). More highly inbred individuals died earlier, usually from mycobacteriosis. This pattern is driven in part by an apparent difference in lifespan between historical versus recent captives, but this effect of genetic background was non‐significant in the multivariate model (Table S3). This tendency in the genetic data, however, does reflect the larger trend of WWWD born more recently tending to die younger (Figure S8). However, the two recent captives with the longest lifespans were the least inbred, supporting inbreeding as the cause of reduced lifespan (Figure 2C), and suggesting that the broader trend of decreased lifespan through time in the studbook data is the result of the cumulative effects of inbreeding depression.

### 3.4 MHC Diversity Is Low in Captivity

Given the effect of inbreeding on susceptibility to mycobacteriosis, we next examined genetic diversity in the major histocompatibility complex, a part of the genome with a key role in immunity broadly and TB specifically (Bettencourt et al. 2020; Witt 2023). By comparing the depths of reads aligned to a single MHC copy and the overall chromosome we estimated MHC gene copy numbers for WWWDs. Coverage of reads mapped to a single MHC copy was different among populations, especially for MHC Class I (Table S4). Based on these differences in relative sequencing depth, we estimate that wild Indonesian WWWDs have at least three MHC class I copies, and 1–2 class IIβ copies. In contrast, captive ducks have approximately half as many copies of class I (1–2), but a comparable number of class IIβ copies.

**FIGURE 3:**
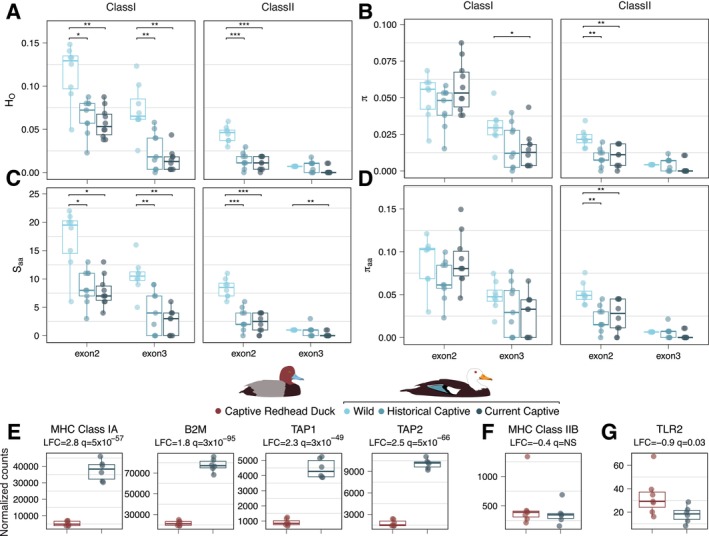
White‐winged wood ducks in captivity have lost nucleotide and amino acid diversity in Major Histocompatibility Complex genes, and show potential immune gene dysregulation. (A–D) Diversity of peptide binding exons in the Major Histocompatibility Complex (MHC). Estimates based on aligning individual data to single exon sequences for each class. * *p* < 0.05, **p < 0.01, ***p < 0.001 (A) Per‐site heterozygosity, (B) Nucleotide diversity, (C) Number of variable amino acids, (D) Pairwise amino acid diversity. Reductions in MHC Class IIB diversity are less dramatic when aligned to the tufted duck genome (Figure S7). (E) MHC Class I and other key genes in the Class I antigen presentation pathway are significantly upregulated in WWWD compared to non‐susceptible RH ducks. (F) MHC Class II is not differentially expressed between these species. (G) Among other differentially expressed immune genes, toll‐like receptor 2 is downregulated in WWWD. For all boxplots, the line is median, the box is the interquartile range (Q1–Q3) and whiskers are +/− 1.5*interquartile range.

There was a significant loss of heterozygosity in the peptide binding region (exons 2–3) of MHC class I genes in captive birds (Figures 3A and S9), with a non‐significant loss in nucleotide diversity (Figures 3B and S9). These patterns are similarly reflected in patterns of amino acid diversity (Figure 3C,D), suggesting a loss of functional diversity. MHC reads aligned to single gene copy suggest a reduction in MHC Class II PBR (exon 2) heterozygosity and nucleotide diversity (Figure 3A), but this was not evident when the data were aligned to the tufted duck genome (Figure S9), perhaps due to variation in read depth across paralogs (Figure S10). Importantly, wild birds have relatively high MHC diversity at both Class I and Class II loci.

### 3.5 Immune Gene Expression in White‐Winged Wood Ducks

To provide a window into the mechanism of susceptibility, we conducted RNAseq on whole blood from WWWDs and closely‐related redhead (RH) ducks *Aythya americana* (Figures S11–S12), which rarely become sick from TB exposure. We found that 56% of the whole‐blood transcriptome was differentially expressed between asymptomatic redhead and asymptomatic WWWDs (5797 genes, *q* < 0.05, Table S5). There were no GO Biological Processes or KEGG pathways that were significantly enriched among these genes (Tables S6 and S7). There were 341 candidate immune genes differentially expressed between species, which was not a significant enrichment (*p* = 0.9). These immune genes included 27 RIG‐I‐like receptor signaling genes (ko04622), and 51 genes in the Tuberculosis KEGG pathway (ko05152) (*p* > 0.05, Table S7). Among the most highly expressed immune genes with strong fold‐change differences were four key MHC genes (Figure S13). These were MHC Class I⍺ and beta‐2 microglobulin (B2M), which dimerize to form the Class I receptor protein, and transporter associated with antigen processing 1 and 2 (TAP1 and TAP2), which together form the antigen presentation complex. All four of these genes are substantially upregulated in asymptomatic WWWDs relative to redhead ducks (LFC = 1.8–2.8, *q* < 5 × 10^−57^, Figure 3E). Within the TB pathway, there was no evidence for differential expression in MHC IIβ (Figure 3F), but toll‐like receptor 2 (TLR2) was downregulated in WWWDs (Figure 3G).

Although limited in sample size, we also contrasted symptomatic and asymptomatic WWWDs. In total, 186 genes were differentially expressed after excluding genes with expression patterns affected by outliers (q* < 0.05; Table S8). There was significant enrichment for genes in the GO Biological Process “Regulation of Apoptotic Process” (GO:0042981, *q* = 0.04, 5 genes). There was also weaker, non‐significant enrichment of genes involved in “Immune Response” (GO:0006955, *q* = 0.1, 5 genes; Table S9), *Yersinia* Infection (ko05135, 5 genes, *p* = 0.03, *q* = 0.8), and Chemokine Signaling Pathway (ko04062, 5 genes, *p* = 0.04, *q* = 0.8; Table S10). We found fewer disease‐related DEGs in redhead ducks (34 genes q* < 0.05) (Table S11), with significant enrichment only for non‐immune KEGG “Replication and Repair” (ko03030, 3 genes, Tables S12 and S13). We found seven immune genes had opposite expression direction in symptomatic individuals of the different species (Figure S14). No genes with significant differences in disease expression were shared between the two species.

## 4 Discussion

With biodiversity disappearing faster than it can be described, we provide here the first characterization of genetic diversity in a critically endangered bird species. The white‐winged wood duck represents a monotypic genus *Asarcornis* (Gonzalez et al. 2009; Johnson and Sorenson 1999) that historically ranged widely from eastern India to Indonesia. Though subspecies are not currently recognized (Rheindt et al. 2025), Indonesian WWWDs have been considered a separate subspecies (*leucoptera*) by earlier authors (Blyth 1849; Hume and Marshall 1880) based on an apparently greater extent of white on their head and mantle plumage as compared to Indian ducks (nominate) (A. J. Green 1993). We found that captive birds derived from northeast India were substantially genetically differentiated from those sampled in Sumatra (Figure 1). The level of genetic divergence between Indian and Indonesian birds, including reciprocal monophyly of mitochondrial lineages, is consistent with the recognition of distinct subspecies. Given our sparse geographic sampling, required by the extreme rarity of these birds, the phylogeographic distribution of these lineages and whether and where there is a clear break between them remains unresolved. Further work is needed to test for associations between genetic and phenotypic variation. Sampling of wild birds in India, where the largest extant populations remain, would provide a more complete picture of genetic diversity patterns in this species.

We assessed genetic variation in WWWD populations, including temporal samples of India‐derived captive birds in the US and wild birds sampled in Indonesia. All three WWWD populations had extremely low genetic diversity with levels similar to other critically endangered species (Table 1) like the five kiwi lineages (*Apteryx* genus) (Bemmels et al. 2021), crested ibis *Nipponia nippon* (Li et al. 2014), and the channel island fox *Urocyon littoralis* (Robinson et al. 2016). Unexpectedly, a sample of wild Indonesian WWWD collected in 1999–2001 had similarly low genetic diversity to the captive populations sourced from India, which have been affected by both founder effects and many generations of inbreeding in captivity. Low genetic diversity in the wild appears to be a result of long‐term population decline driven by environmental change during the Pleistocene (Figure 2), and perhaps compounded by founder effects during colonization of Sumatra and/or subsequent isolation following sea‐level rise and insularization (Husson et al. 2020). Therefore, the low observed genetic diversity in the wild is not solely driven by recent anthropogenic effects, but signatures of recent inbreeding are likely signals of recent fragmentation (Khan et al. 2021). Instead, environmental change during Pleistocene glacial cycles and the associated reduction of forest habitats (Louys and Roberts 2020) appear to have initiated the decline of WWWDs globally. This observed demographic trajectory of WWWDs is similar to other tropical vertebrates (Gu et al. 2023; Karjee et al. 2025), including the critically endangered Sumatran rhino, which shares a similar geographic distribution and habitat requirements (von Seth et al. 2021). As a charismatic megafauna species, the Sumatran rhino is a “flagship” for conservation efforts that may also promote the persistence of WWWDs.

**TABLE 1:** Nucletotide diversity estimates based on genome‐wide surveys of birds of conservation concern. Colored cells highlight WWWDs and correspond to population color scheme used throughout.

Common name	Scientific name	Nucleotide diversity	References
Brown eared pheasant—W lineage	*Crossoptilon mantchuricum*	0.0001	Wang et al. (2021)
Brown eared pheasant—C lineage	*Crossoptilon mantchuricum*	0.0002	Wang et al. (2021)
Green peafowl—museum	*Pavo muticus*	0.0005	Dong et al. (2021)
Raso lark	*Alauda razae*	0.0005	Dierickx et al. (2020)
Green peafowl—modern	*Pavo muticus*	0.0005	Dong et al. (2021)
Spotted kiwi	*Apteryx owenii*	0.0007	Bemmels et al. (2021)
**White‐winged duck—captive/modern**	*Asarcornis scutulata*	0.0008	This study
**White‐winged duck—wild/Indonesian**	*Asarcornis scutulata*	0.0010	This study
Brown kiwi	*Apteryx rowi*	0.0010	Bemmels et al. (2021)
Oriental stork‐wild	*Ciconia boyciana*	0.0010	Yang et al. (2024)
Oriental stork‐captive	*Ciconia boyciana*	0.0010	Yang et al. (2024)
Brown kiwi	*Apterx australis*	0.0015	Bemmels et al. (2021)
White‐winged duck—captive/historical	*Asarcornis scutulata*	0.0015	This study
Spotted kiwi	*Apteryx haastii*	0.0015	Bemmels et al. (2021)
Brown kiwi	*Apteryx mantelli*	0.0018	Bemmels et al. (2021)
Lesser prairie‐chicken	*Tympanuchus pallidicinctu*	0.0036	Black et al. (2024)
Greater prairie‐chicken	*Tympanuchus cupido*	0.0039	Black et al. (2024)

The current captive US population of WWWDs is derived from a single pair of founders from Assam, India. As a result, we observed that genetic diversity has eroded substantially over time and that more highly inbred birds had shorter lifespans (Figure 2C). This pattern is largely explained by differences in lifespan between individuals in our current and historical samples, and so could be explained in part by possible increases in TB exposure over time. Among our “current” sample, however, the two birds with the longest lifespans were the least inbred, suggesting that inbreeding depression, and not just TB exposure, contributes to reduced lifespans. This finding suggests that genetic improvements to the current captive stock will be an important part of future WWWD *ex situ* management (Humble et al. 2023). Most WWWDs breed at ~3 years of age, and early in the breeding program, birds lived long enough to lay multiple clutches of eggs. With birds now dying younger, this is no longer the case (Cook 2016).

Despite having low overall genetic diversity, wild Indonesian WWWDs had higher MHC diversity than the captive population (Figure 3A–D), representing a potentially important reservoir of functional genetic variation. Similar retention of MHC diversity has been seen in other bird species that have suffered population bottlenecks during island colonization (Newhouse and Balakrishnan 2015; Stervander et al. 2020). The observed difference in MHC diversity between captive Indian and wild Indonesian birds is due to an apparent difference in copy number, with further reductions in per locus diversity through time. Due to our sampling, however, it remains unclear whether the difference in copy number is an evolved difference between Indian and Indonesian birds, or is due to the two US founders from the Indian population having an atypically small number of loci. Sampling of wild Indian birds would resolve this uncertainty.

Given the low MHC diversity in captive birds, we sought further insights into the mechanism driving differences in susceptibility between WWWDs and other birds housed in the same conditions. By conducting RNAseq on a small number of asymptomatic WWWDs and closely related redhead ducks, we identified extensive gene expression differences between these species. Given these extensive differences, however, inferences on the mechanisms of susceptibility are speculative. Nevertheless, we note substantial differences in the expression of immune genes, including many genes associated with the RIG‐I pathway, a critical component of innate immune response to TB (Skvortsova et al. 2023), and the human tuberculosis KEGG pathway. One TB pathway gene, TLR2, is down‐regulated in asymptomatic WWWDs relative to redhead ducks. TLR2 plays a critical role in the innate immune response. In mice, exposure to *Mycobacterium tuberculosis* downregulates TLR2 and, in turn, interferes with the induction of the type I interferon response and cross processing by MHC Class I (Simmons et al. 2010; Witt 2023). *M. tuberculosis* itself produces TLR2 agonists in mice. We speculate that *M. avium* may have a similar effect in WWWD, whereas redhead ducks may be resistant to this form of immune system evasion. Necropsies of WWWDs have revealed broad dissemination of the disease across tissues, a high concentration of mycobacteria, and a lack of multinucleated giant cells within lesions, all suggesting a failure of cell mediated immunity (Saggese et al. 2007). MHC Class I, its heterodimer B2M, and associated transport proteins TAP1 and TAP2 were all strongly upregulated in asymptomatic WWWDs relative to redheads, but no MHC genes were differentially expressed between symptomatic and asymptomatic WWWDs. Differences in MHC expression in combination with reduced MHC diversity in captivity suggest that these loci may be an important consideration in future genetic management.

Our findings strongly suggest that the genetic health of the captive WWWD population in the US is a key factor in their susceptibility to *M. avium* , but perhaps not the only factor. Husbandry changes, such as breeding birds at lower density, may be effective in reducing exposure to shed virus. Until now, vaccine development and administration has been prohibitively expensive, and a previous vaccination effort in the UK was not successful at preventing disease in adult WWWDs that had presumably been exposed to TB prior to vaccination (Cromie 1991). With the advent of mRNA‐based vaccines for TB (Touray et al. 2023), cost‐effective vaccines may be within reach and may be successful if aimed at TB‐naive hatchlings. We suggest that a combination of genetic improvement, improved husbandry, and vaccination should be pursued to improve captive breeding success. These efforts should be coordinated internationally, with an emphasis on working within the native range of the species, and maximizing positive conservation outcomes in the wild.

## Funding

This work was supported by the American Genetics Association and Akron Zoo. East Carolina University, International Wild Waterfowl Association, Raleigh Durham Caged Bird Society, American Federation of Aviculture, Avicultural Society of America, Carolina Virginia Pheasant and Waterfowl Society, NED was supported by Wildlife Conservation Society, a David Boren Fellowship, and a Fulbright Scholarship.

## Conflicts of Interest

The authors declare no conflicts of interest.

## Supporting information


**Figure S1:** Time calibrated mtDNA phylogeny of ducks including Indian‐derived and Indonesian White‐winged wood ducks ( *Asarcornis scutulata* , top).
**Figure S2:** Demographic history constructed using PSMC.
**Figure S3:** Historical captive birds contain more diversity than wild birds in Indonesia and current captive birds.
**Figure S4:** Inbreeding coefficients from genome‐wide heterozygosity using ngsF (left) and fraction of genome in ROHs (right).
**Figure S5:** Model assumptions checks from DHARMa indicating no significant departures from normality of residuals comparing inbreeding coefficient and lifespan.
**Figure S6:** Weak negative relationship between F_ROH_ inbreeding coefficient and lifespan.
**Figure S7:** Model assumptions checks from DHARMa indicating no significant departures from normality of residuals comparing F_ROH_ and lifespan.
**Figure S8:** Decrease in lifespan by sex through time of the US captive population (top), which showed a significant effect of bird year but not sex in quantile regression (bottom).
**Figure S9:** Heterozygosity and nucleotide diversity of MHC Peptide binding sequences when aligned to the tufted duck genome.
**Figure S10:** SNP depth and density is inconsistent across MHC class II copies in the tufted duck genome.
**Figure S11:** Number of whole‐blood RNAseq reads mapped to the tufted duck genome.
**Figure S12:** PCA plot of all RNAseq samples after minimum count filtering.
**Figure S13:** MA‐plot of healthy duck RNAseq data
**Figure S14:** Comparing disease expression profiles between redhead and WWWDs.

**Table S1:** DNA Samples, GenBank Accession Numbers and sequencing data.
**Table S2:** RNA Samples and metadata.
**Table S3:** Statistical model for the relationship between age and inbreeding coefficient.
**Table S4:** MHC copy number estimates.
**Table S5:** Differential expression statistics for Redhead versus WWWDs.
**Table S6:** Gene Ontology enrichment analysis of differentially expressed genes in Redhead vs. WWWDs.
**Table S7:** KEGG enrichment analysis of differentially expressed genes in Redhead vs. WWWDs.
**Table S8:** Differential expression statistics for healthy versus infected WWWDs.
**Table S9:** Gene Ontology enrichment analysis of differentially expressed genes in healthy versus infected WWWDs.
**Table S10:** KEGG enrichment analysis of differentially expressed genes in healthy versus infected WWWDs.
**Table S11:** Differential expression statistics for healthy versus infected redhead ducks.
**Table S12:** Gene Ontology enrichment analysis for healthy versus infected redhead ducks.
**Table S13:** KEGG enrichment analysis for healthy versus infected redhead ducks.

## Data Availability

Raw DNA sequence data are available under PRJNA1258233 (Table S1), raw gene expression data are available under PRJNA1285963 (Table S4). All data and code are provided in Dryad: https://datadryad.org/share/W8y3‐WQsDfsm4F9YzkKS1oUhK0d1NrdAfLcKz9XN76I.
