# Supplementary material for: Tuberculosis Susceptibility and Inbreeding Depression Hinder Ex Situ Conservation in a Critically Endangered Rainforest Bird
Source: Evol Appl. 2026 Jun 23;19(6):e70286. doi: 10.1111/eva.70286 (PMC13291423; doi:10.1111/eva.70286)
Supplement: Supplementary file 1 — Figure S1: Time calibrated mtDNA phylogeny of ducks including Indian‐derived and Indonesian White‐winged wood ducks ( Asarcornis scutulata , top). Figure S2: Demographic history constructed using PSMC. Figure S3: Historical captive birds contain more diversity than wild birds in Indonesia and current captive birds. Figure S4: Inbreeding coefficients from genome‐wide heterozygosity using ngsF (left) and fraction of genome in ROHs (right). Figure S5: Model assumptions checks from DHARMa indicating no significant departures from normality of residuals comparing inbreeding coefficient and lifespan. Figure S6: Weak negative relationship between FROH inbreeding coefficient and lifespan. Figure S7: Model assumptions checks from DHARMa indicating no significant departures from normality of residuals comparing FROH and lifespan. Figure S8: Decrease in lifespan by sex through time of the US captive population (top), which showed a significant effect of bird year but not sex in quantile regression (bottom). Figure S9: Heterozygosity and nucleotide diversity of MHC Peptide binding sequences when aligned to the tufted duck genome. Figure S10: SNP depth and density is inconsistent across MHC class II copies in the tufted duck genome. Figure S11: Number of whole‐blood RNAseq reads mapped to the tufted duck genome. Figure S12: PCA plot of all RNAseq samples after minimum count filtering. Figure S13: MA‐plot of healthy duck RNAseq data Figure S14: Comparing disease expression profiles between redhead and WWWDs. [file EVA-19-e70286-s002.docx]

**Supplementary Information and Figures**

**
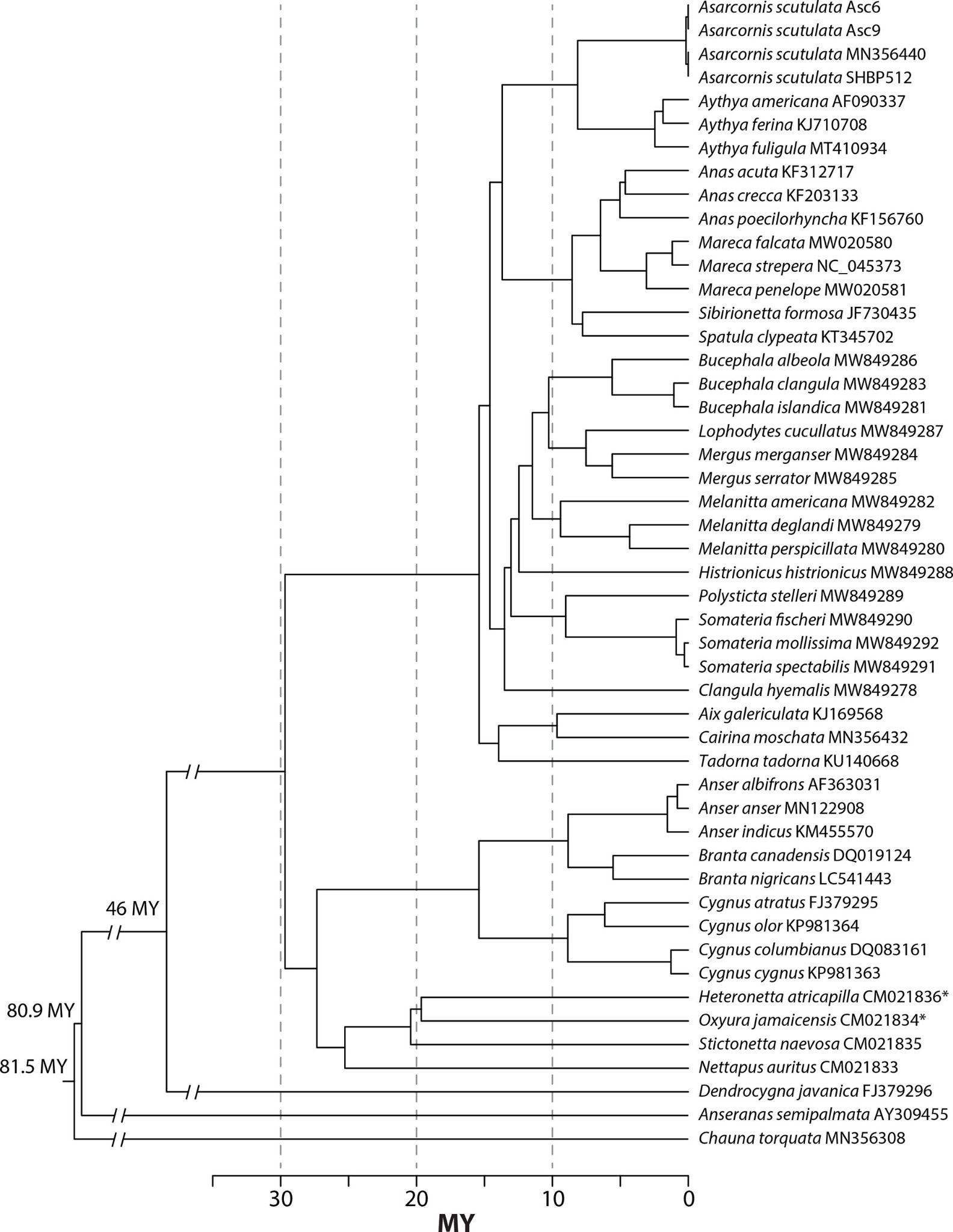
**

**Figure S1:** Time calibrated mtDNA phylogeny of ducks including Indian-derived and Indonesian White-winged wood ducks (*Asarcornis scutulata*, top).


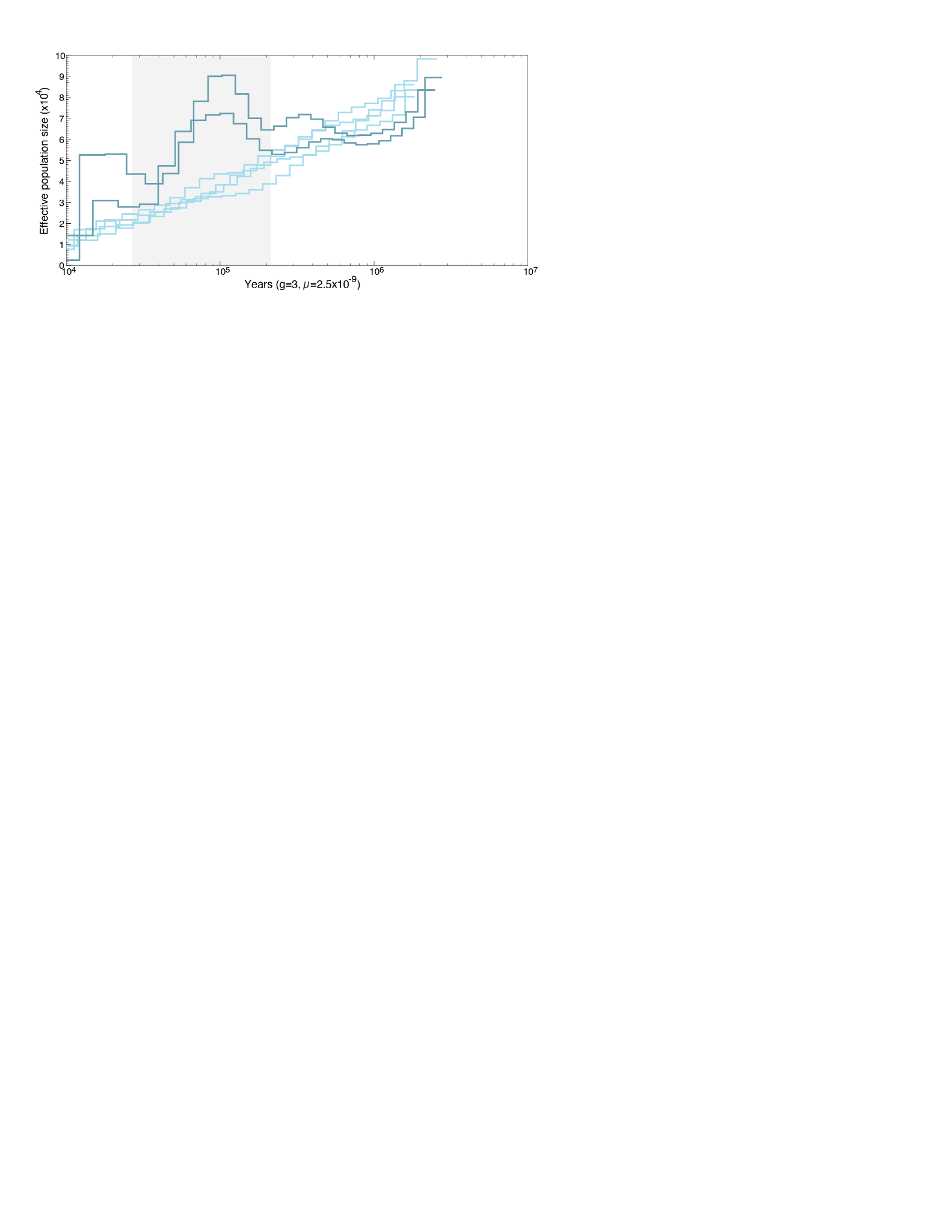


**Figure S2: Demographic history constructed using PSMC**. As in Figure 2A, the grey rectangle and dotted line indicates the Last Glacial Period and the Last Glacial Maximum, respectively. Light blue lines are derived from the Indonesian “wild” population, and darker blue comes from the historical captive sample derived from Assam, India.


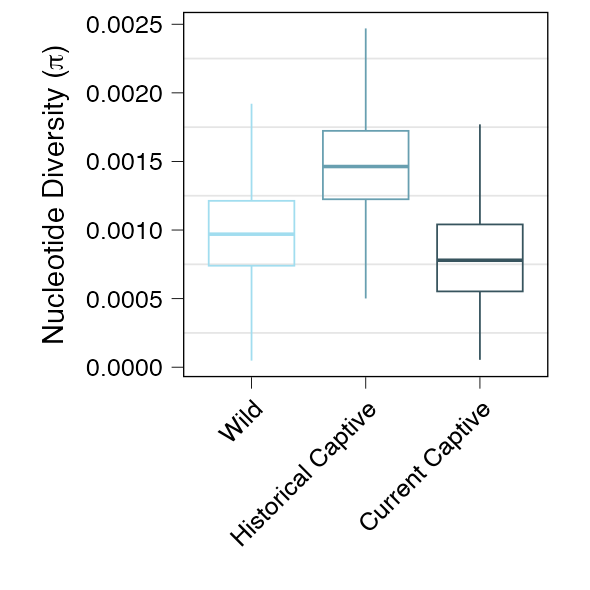

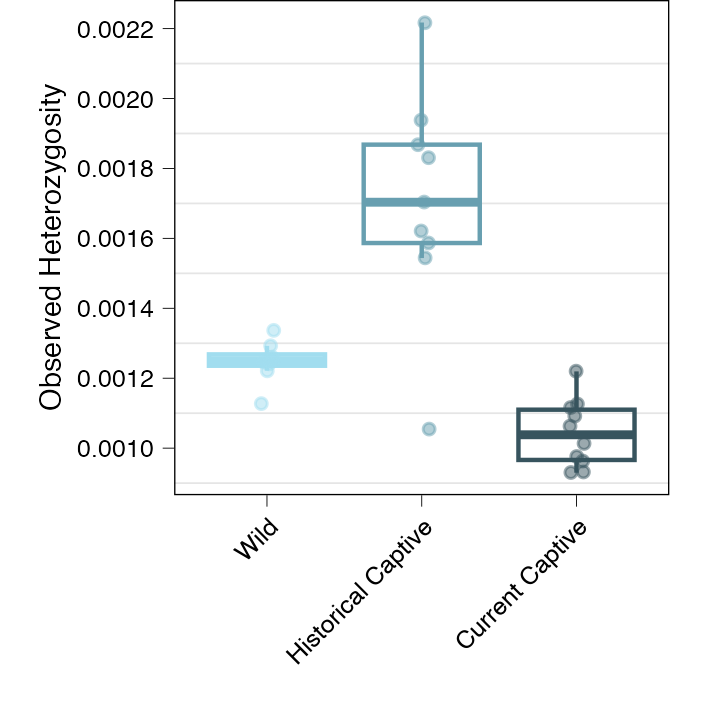


**Figure S3: Historical captive birds contain more diversity than wild birds in Indonesia and current captive birds.**

Left Panel: Nucleotide diversity calculated in 100kb windows across the genome.

Right Panel: Individual heterozygosity calculated using variant and invariant sites, where each point is an individual.


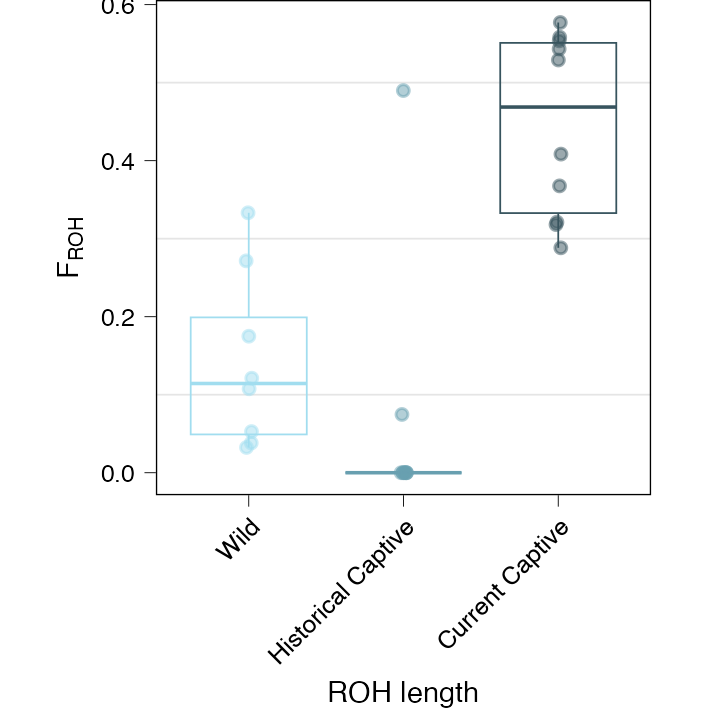

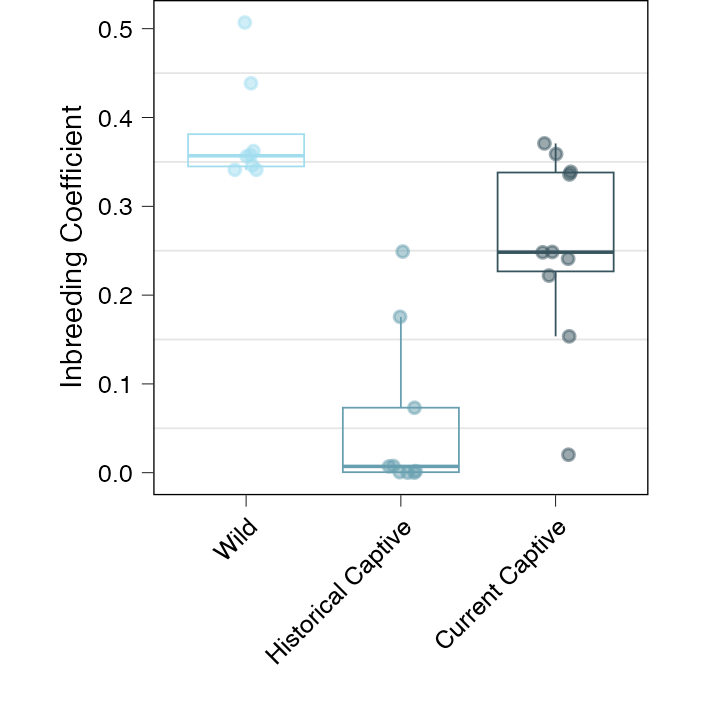


**Figure S4: Inbreeding coefficients from genome-wide heterozygosity using ngsF (left) and fraction of genome in ROHs (right).**

Each point represents the inbreeding coefficient of each individual in the sampled population.


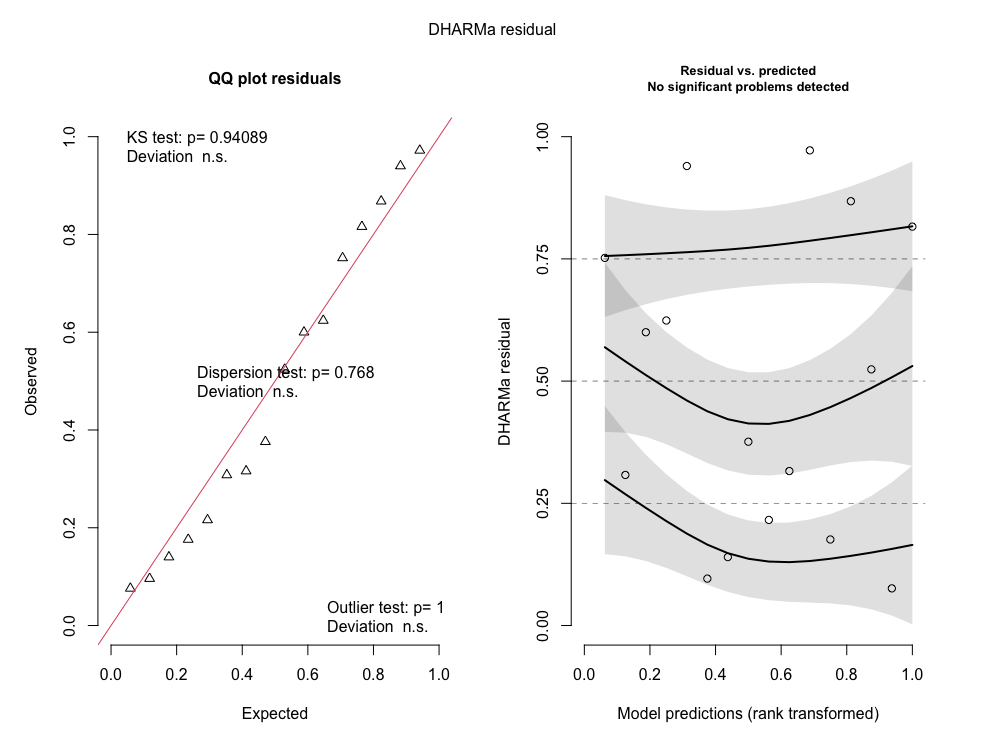


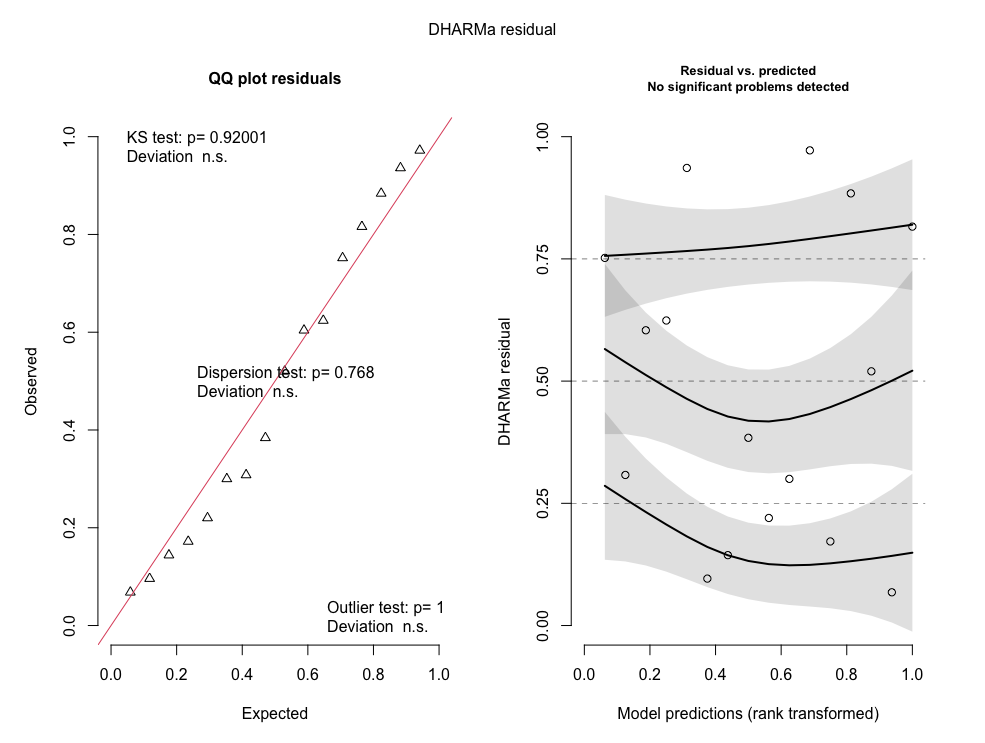


**Figure S5: Model assumptions checks from DHARMa indicating no significant departures from normality of residuals comparing inbreeding coefficient and lifespan. See also Table S2.**

Top model: inbreeding ~ population + age

Bottom model: inbreeding ~ age


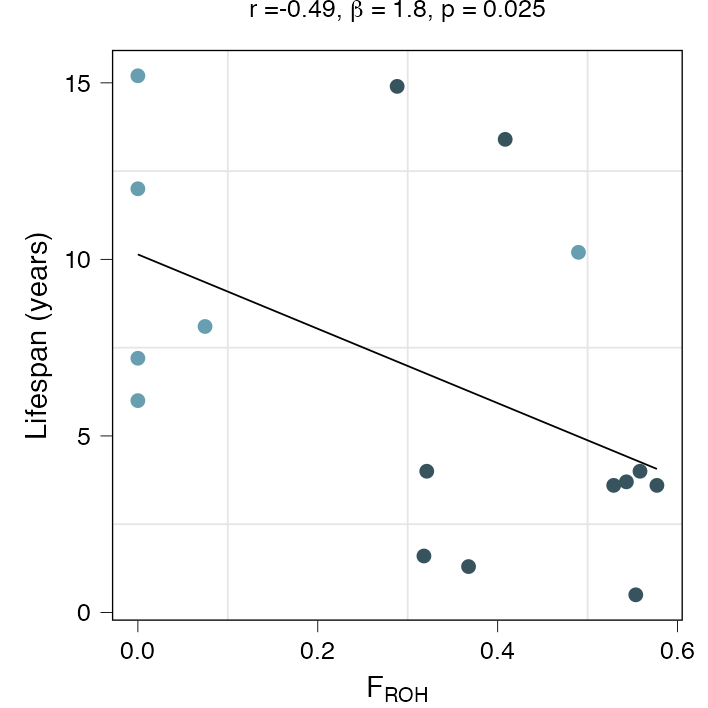


**Figure S6: Weak negative relationship between F_ROH_ inbreeding coefficient and lifespan.**

Fitted line is from model age ~ F_ROH_ (r = -0.5, p(F_ROH_) = 0.025). Table S2 includes models outputs, and is indistinguishable by AIC from the nonsignificant model that includes a population variable. (age ~ pop + F_ROH_: p(F_ROH_) = 0.45).


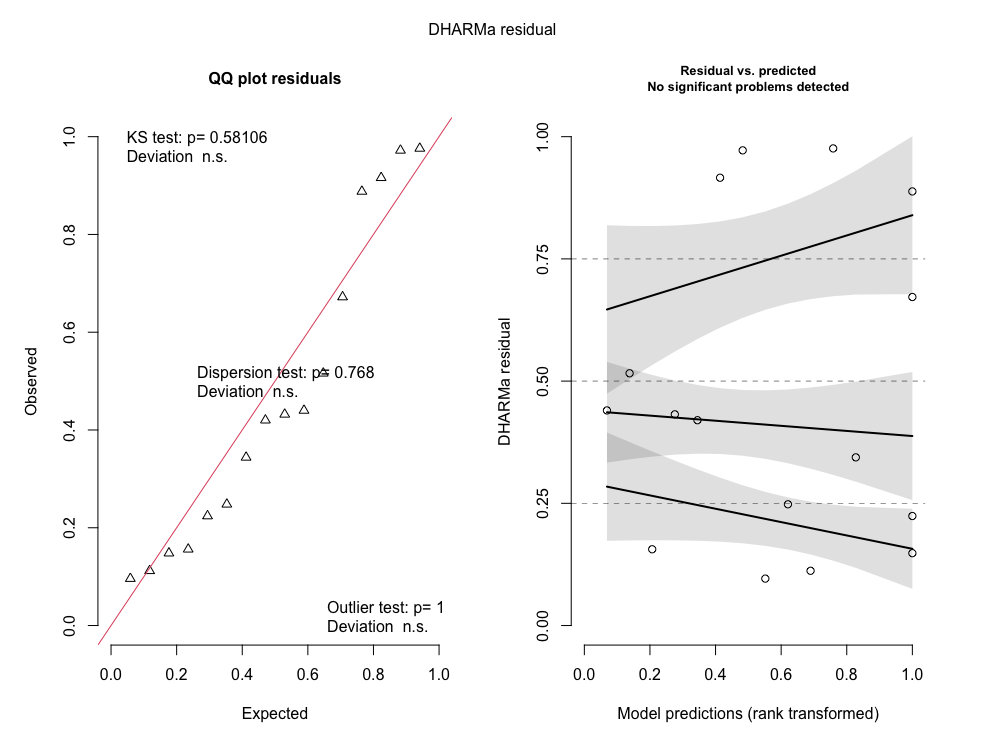


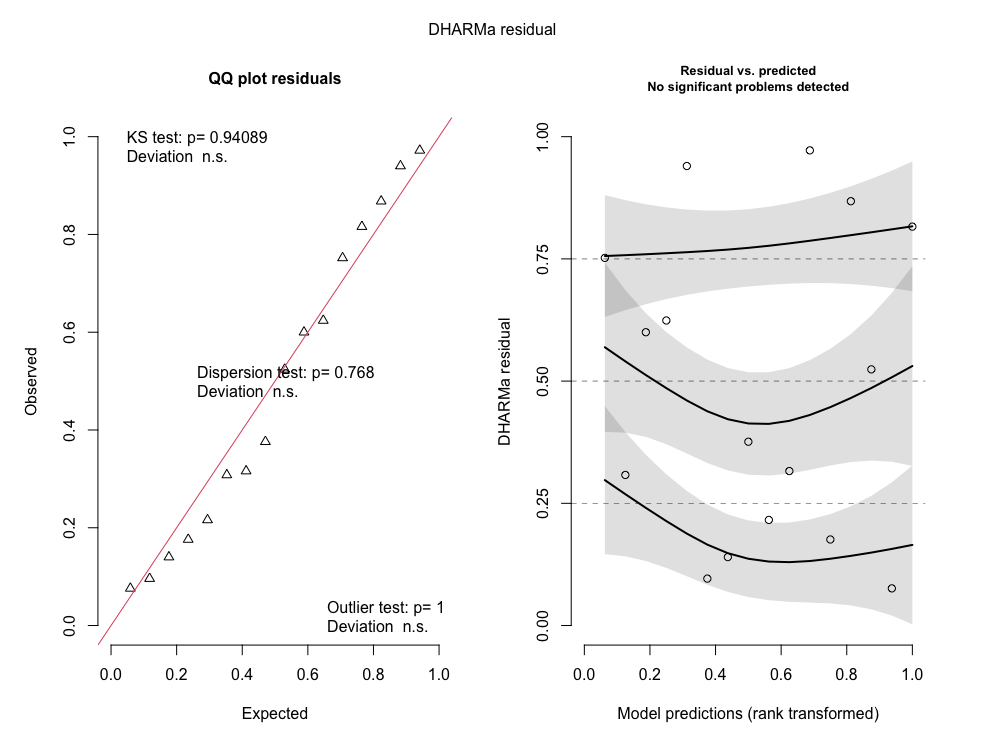


**Figure S7: Model assumptions checks from DHARMa indicating no significant departures from normality of residuals comparing F_ROH_ and lifespan. See also Table S2.**

Top model: F_ROH_ ~ age

Bottom model: F_ROH_ ~ population + age


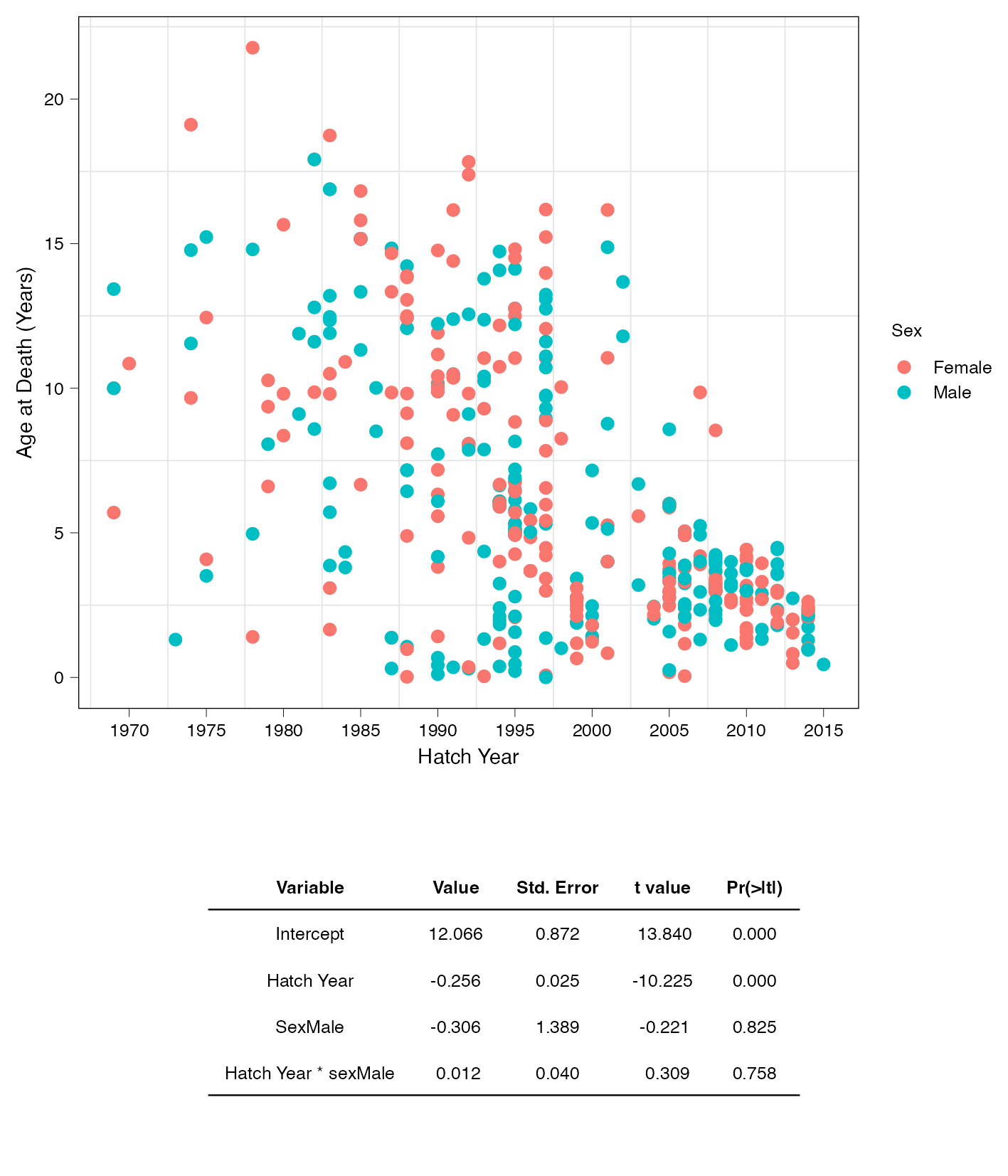


**Figure S8: Decrease in lifespan by sex through time of the US captive population (top), which showed a significant effect of bird year but not sex in quantile regression (bottom).**

Studbook records for larger analysis showing decline in lifespan through time (by Hatch Year) in the US captive population. The quantile regression model form was Age_Death ~ Hatch_Year*Sex. Standard errors, t-values and p-values were calculated by bootstrap resampling 1000 times.


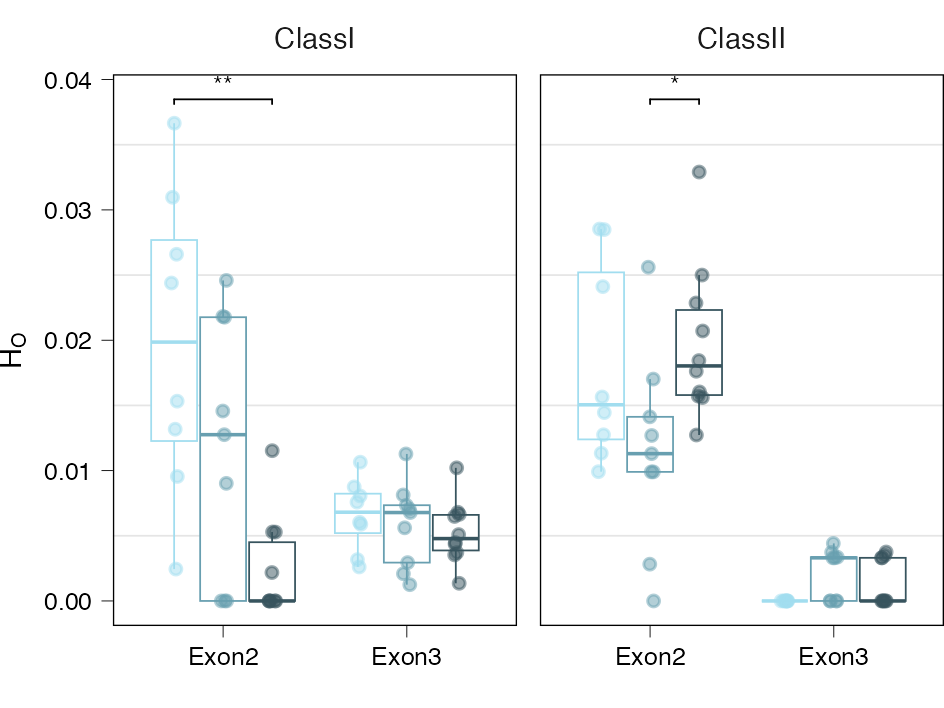


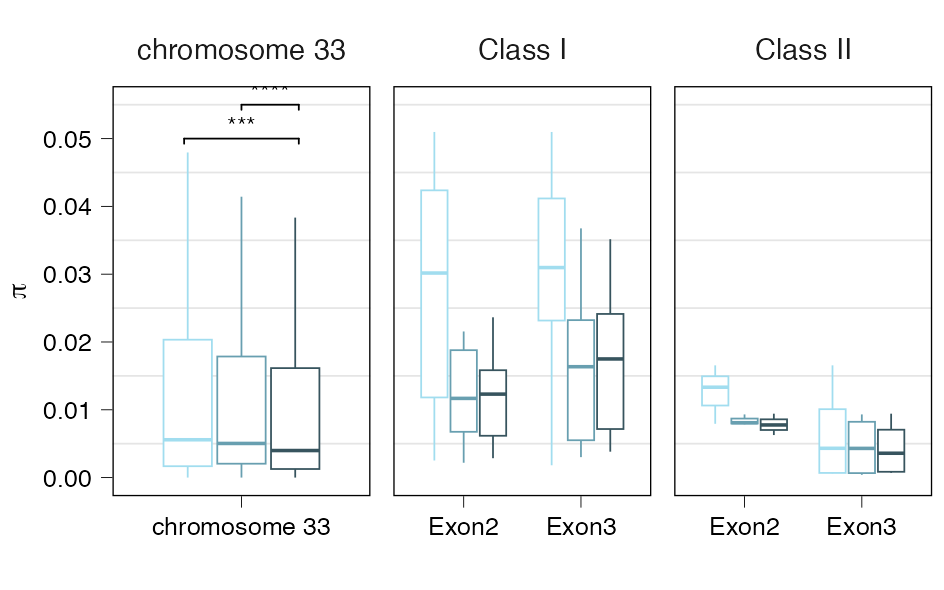


**Figure S9: heterozygosity (top) and nucleotide diversity (bottom) of MHC Peptide binding sequences when aligned to the tufted duck genome.** Colours (left to right/light to dark) are wild, historical captive, and current captive. We estimated heterozygosity directly from called genotypes for each individual as the number of heterozygous sites among variant and invariant sites in the exons. We calculated nucleotide diversity (π) in 1kb windows using pixy. Plots indicate significant wilcoxon tests adjusted for multiple testing with Holm method.


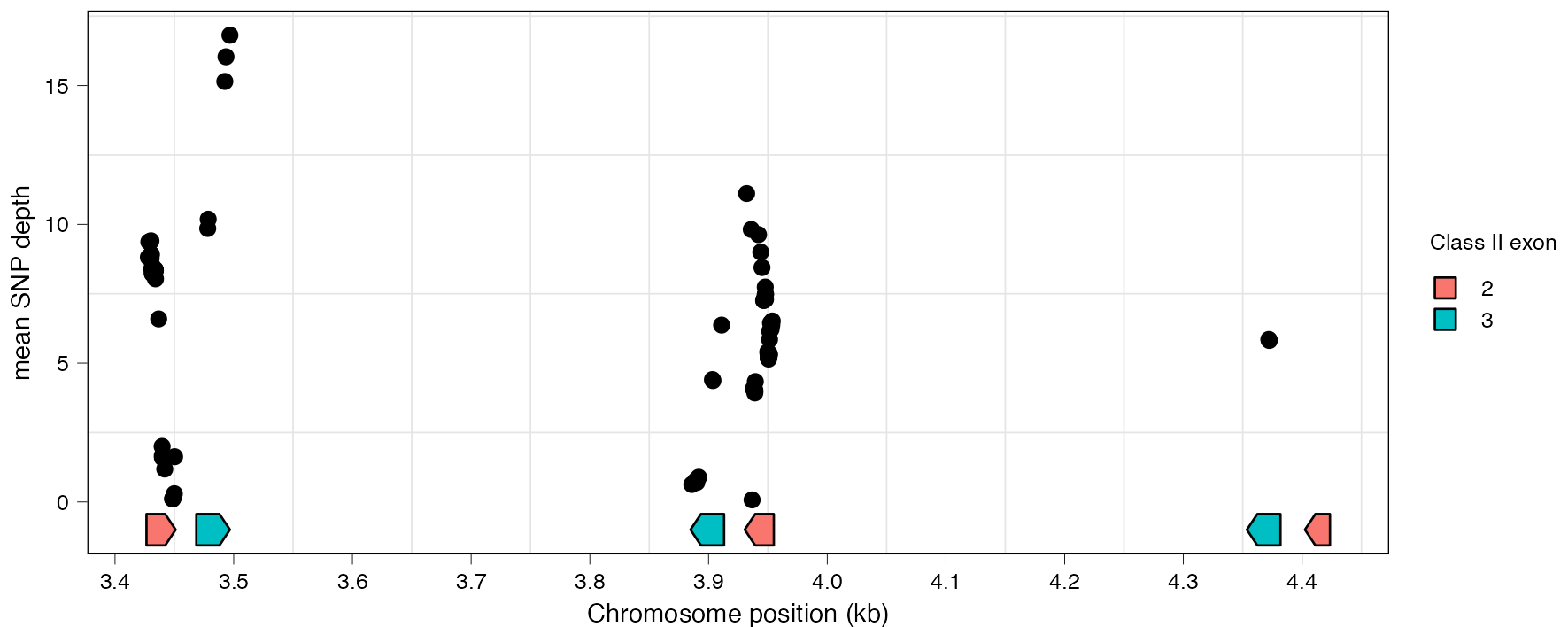


**Figure S10: SNP depth and density is inconsistent across MHC class II copies in the tufted duck genome.**

Figure is annotated with positions of class II exons, where each dot represents a SNP.


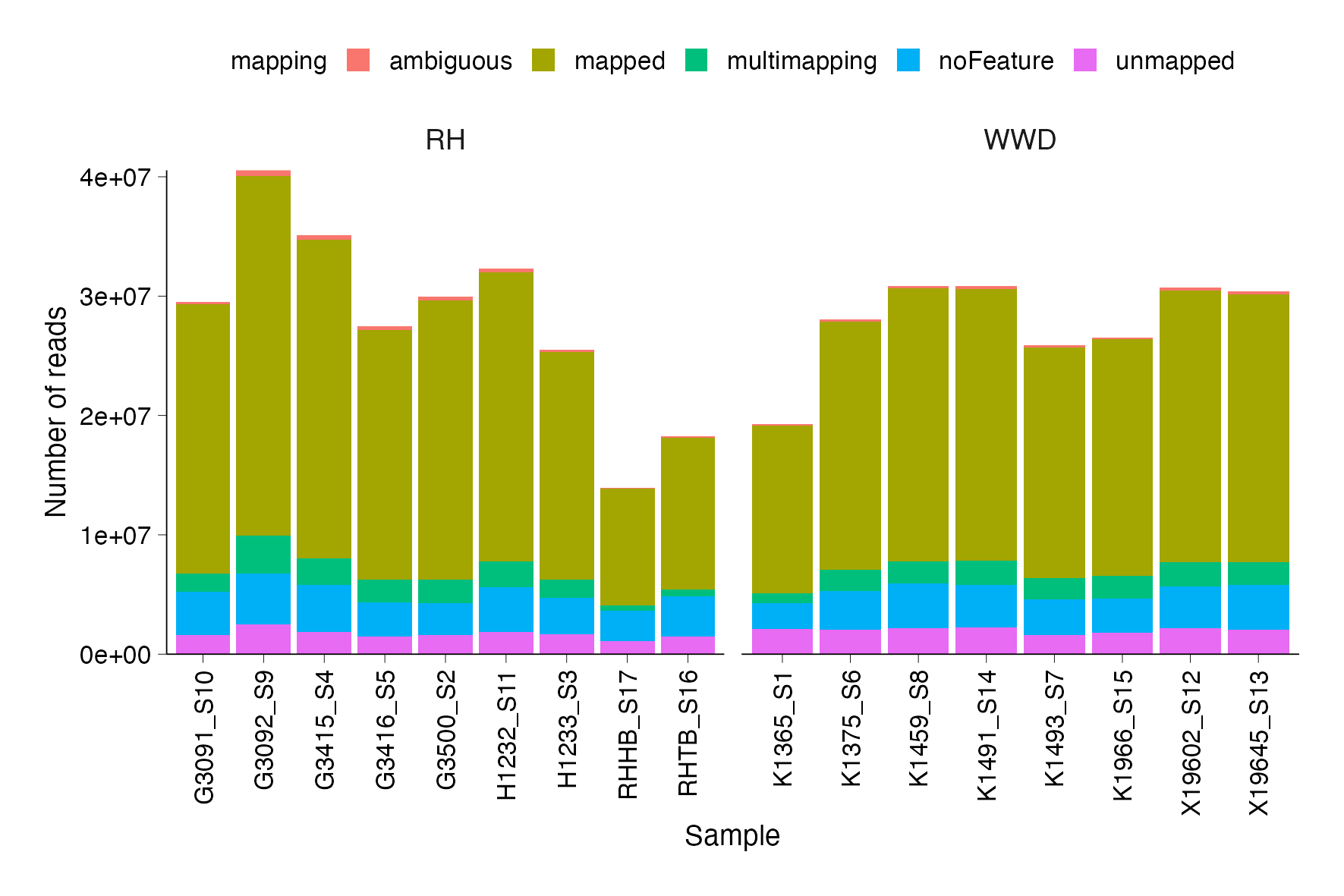


**Figure S11: Number of whole-blood RNAseq reads mapped to the tufted duck genome.**

Each sample is an individual redhead or white-winged duck, and includes the number of reads mapped to gene features in the *A. fuligula* genome (dark mustard colour). Other colours represent mapping flags and number of reads that were not included in downstream analyses.

**
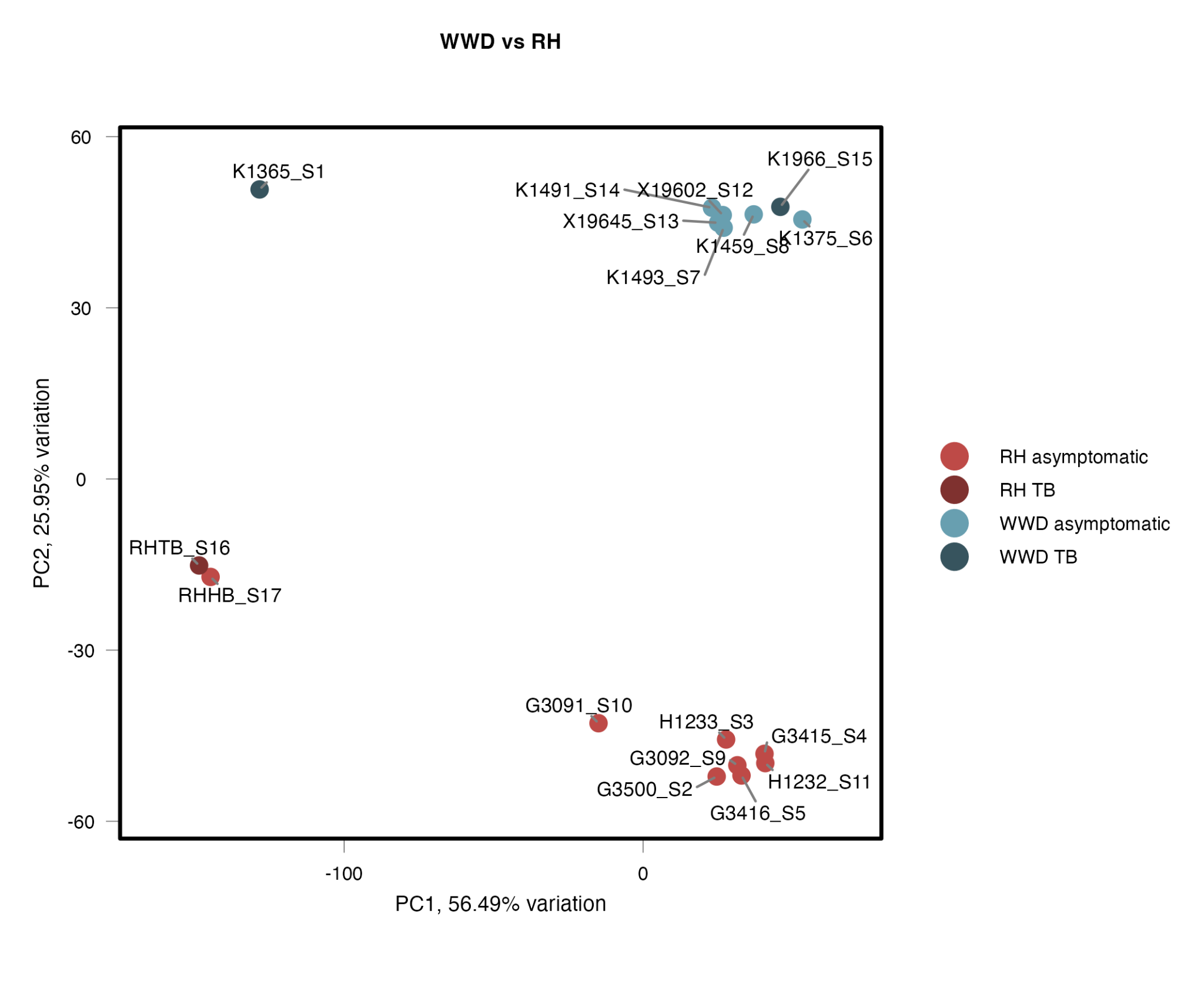
Figure S12: PCA plot of all RNAseq samples after minimum count filtering.**

Each point is an individual’s library, where colour represents species and point darkness indicates individuals that were symptomatic for mycobacteriosis (TB).


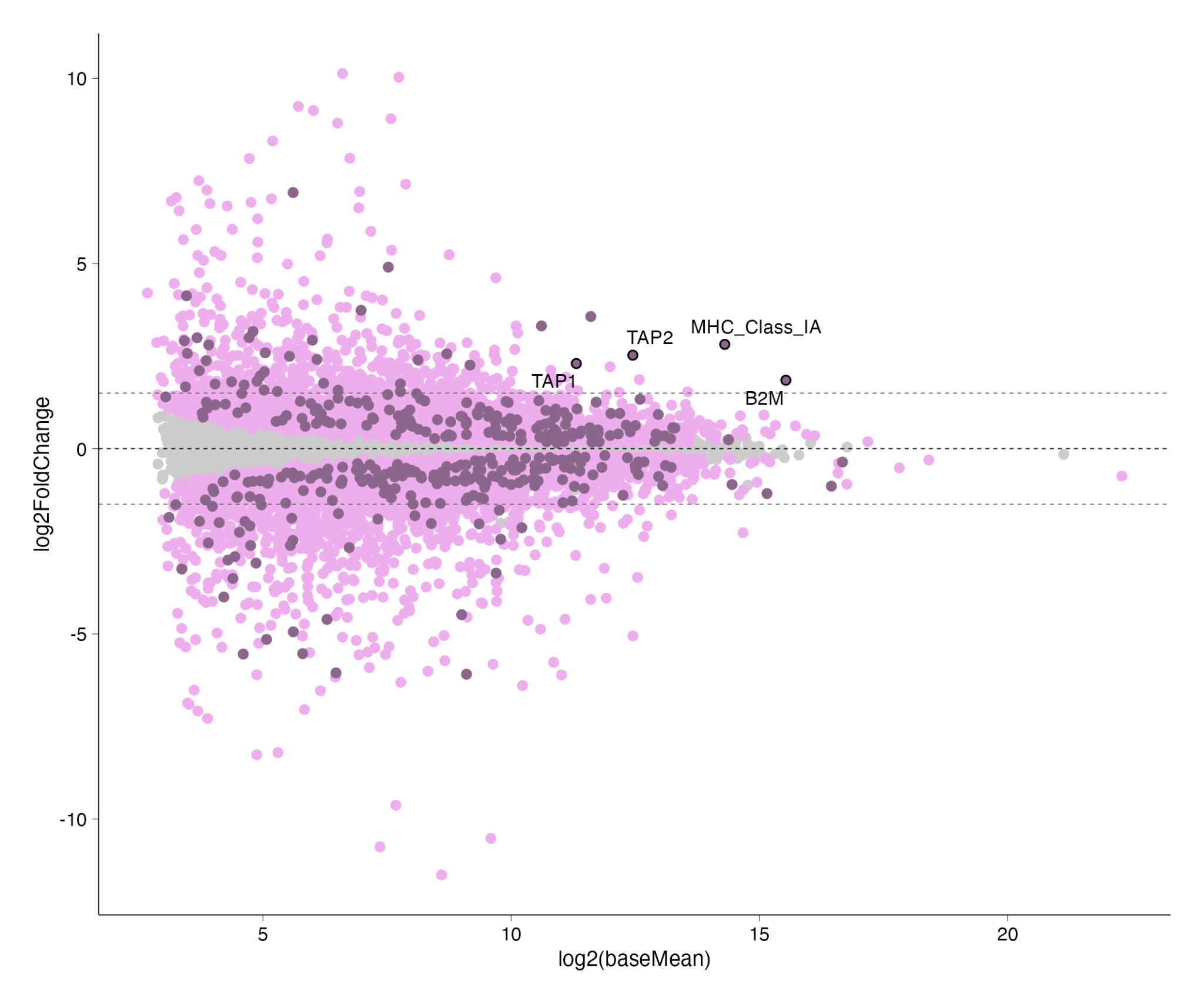


**Figure S13: MA-plot of healthy duck RNAseq data**

The X-axis shows the log2 transformed average of the normalized count for each gene, with its corresponding log2 fold change difference between species, where a positive value indicates higher expression in healthy white-winged ducks. A log2 fold change threshold of expression (|1.5|) is annotated in light grey lines, and no difference in expression is indicated at zero with a black line. Light pink points are all significantly differentially expressed genes (*q* < 0.05) and the darker pink points indicate genes we annotated in the tufted duck immune system. Labelled genes indicate the MHC Class I complex genes that have strong fold changes, high levels of expression, and highly significant (Figure 3).


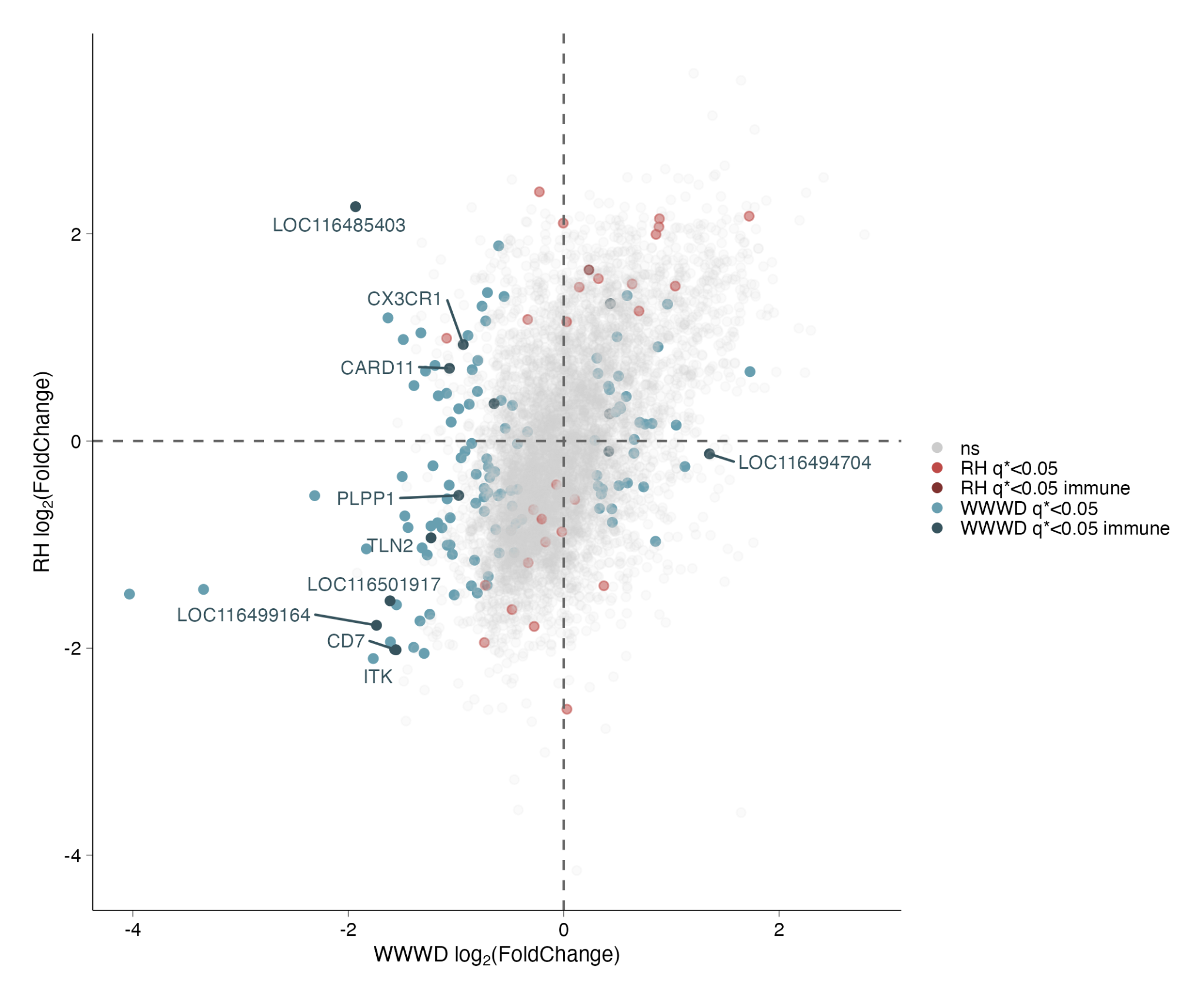


**Figure S14: Comparing disease expression profiles between redhead and WWWDs.**

The X-axis shows the direction and magnitude of expression of genes in symptomatic WWWDs, where positive values indicate upregulated in symptomatic individuals compared to asymptomatic individuals. Y-axis shows direction and magnitude of expression of genes in symptomatic redhead (RH) ducks. Genes that lie in bottom left and top right of the plot show similar expression profiles between species, while genes occupying the other quadrants show genes that are expressed differently in sick birds of these species. Coloured points are genes that were significant in each species’ comparison and darker coloured points indicate immune system genes.
